# ALIX Regulates Tumor-Mediated Immunosuppression by Controlling EGFR Activity and PD-L1 Presentation

**DOI:** 10.1016/j.celrep.2026.117882

**Published:** 2026-08-08

**Authors:** James Monypenny, Hanna Milewicz, Fabian Flores-Borja, Gregory Weitsman, Anthony Cheung, Ruhe Chowdhury, Thomas Burgoyne, Appitha Arulappu, Katherine Lawler, Paul R. Barber, Jose M. Vicencio, Melanie Keppler, Wahyu Wulaningsih, Sean M. Davidson, Franca Fraternali, Natalie Woodman, Mark Turmaine, Cheryl Gillett, Dafne Franz, Sergio A. Quezada, Clare E. Futter, Alex Von Kriegsheim, Walter Kolch, Borivoj Vojnovic, Jeremy G. Carlton, Tony Ng

## Main text

(Cell Reports *24*, 630–641; July 17, 2018)

As published, blots showing the PD168393-sensitive, EGF-dependent phosphorylation of the CrkII-based Picchu-X biosensor in HCC1954 cells (Figure 1A) were inadvertently duplicated from an experiment showing the Mo-IPQA-sensitive, EGF-dependent phosphorylation of the CrkII-based Picchu-X biosensor in HCC1954 cells. This latter experiment was published as Figure 1B in PMID: 28166195.

Mo-IPQA is a PD168393 derivative, and figures for both papers were prepared concurrently, leading to the inadvertent duplication.

The corrected panel, which can be seen below, now displays the originally acquired blots showing PD168393-sensitive, EGF-dependent phosphorylation of the CrkII-based Picchu-X biosensor in HCC1954 cells.

There is no change to the interpretation of the data in either manuscript. The authors apologize for any confusion this may have caused.

**Figure 1A:**
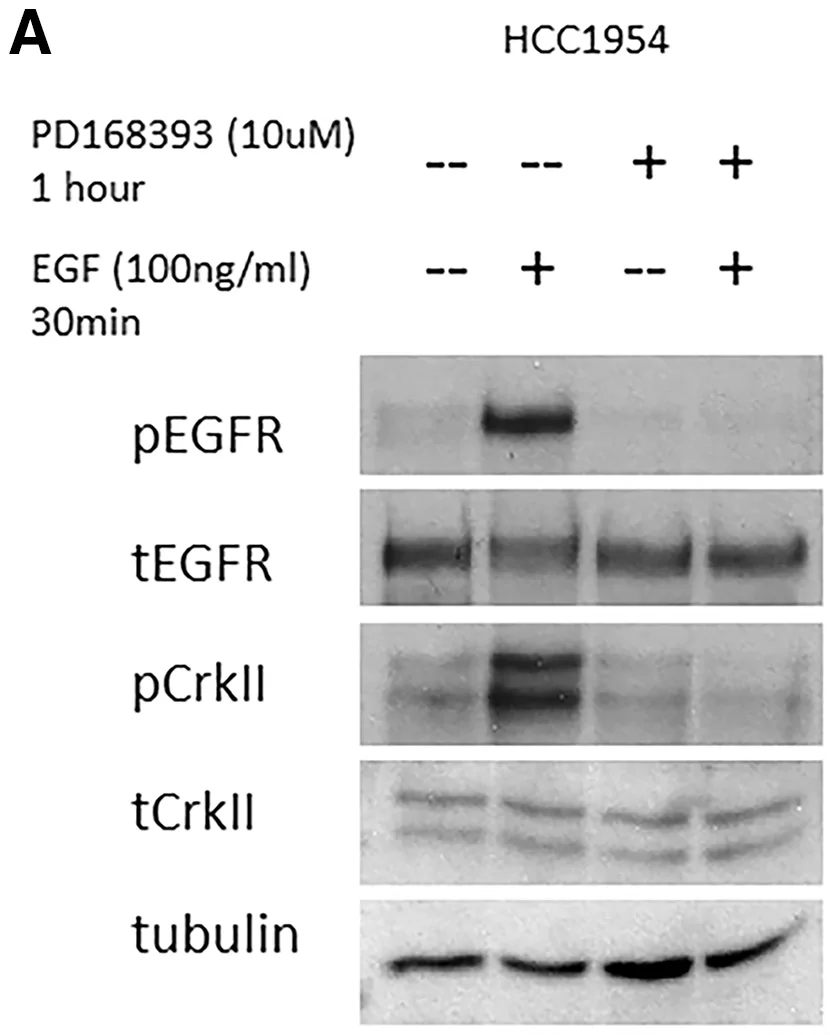
(corrected)

