# Supplementary material for: ALIX Regulates Tumor-Mediated Immunosuppression by Controlling EGFR Activity and PD-L1 Presentation
Source: Cell Rep. 2018 Jul 17;24(3):630–41. doi: 10.1016/j.celrep.2018.06.066 (PMC6077252; doi:10.1016/j.celrep.2018.06.066)
Supplement: Document S1. Supplemental Experimental Procedures, Figures S1–S6, and Tables S1–S4 [file mmc1.pdf]

Cell Reports, Volume *24*

Supplemental Information

ALIX Regulates Tumor-Mediated Immunosuppression by Controlling EGFR Activity and PD-L1 Presentation

James Monypenny, Hanna Milewicz, Fabian Flores-Borja, Gregory Weitsman, Anthony Cheung, Ruhe Chowdhury, Thomas Burgoyne, Appitha Arulappu, Katherine Lawler, Paul R. Barber, Jose M. Vicencio, Melanie Keppler, Wahyu Wulaningsih, Sean M. Davidson, Franca Fraternali, Natalie Woodman, Mark Turmaine, Cheryl Gillett, Dafne Franz, Sergio A. Quezada, Clare E. Futter, Alex Von Kriegsheim, Walter Kolch, Borivoj Vojnovic, Jeremy G. Carlton, and Tony Ng

Supplemental figures

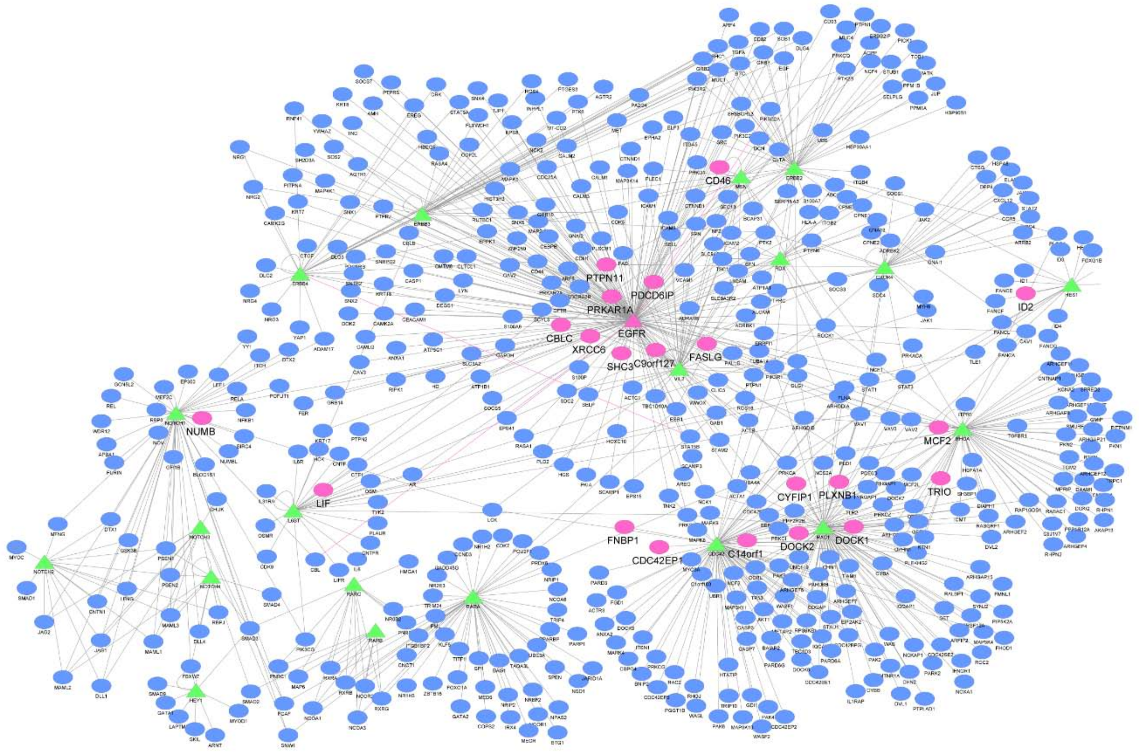

Figure S1. The EGFR protein interaction network, Related to Figure 2.

Visual representation of the EGFR protein interaction network generated using Cytoscape software (The Cytoscape Consortium: http://www.cytoscape.org/). The network was designed using the Human Protein Reference Database (HPRD, http://www.hprd.org/) to predict potential targets that contribute to a malignant phenotype in breast cancer. The HPRD was mined for proteins within an EGFR-containing protein subnetwork which included the EGFR family members (EGFR, HER2, HER3 and HER4), as well 16 other seed proteins from our previously published sub-network constituting membrane receptors, protein kinases, and cytoskeletal proteins associated with a cancer 'metastatic' phenotype (Fruhwirth et al., 2011; Weitsman et al., 2014). These proteins were used as seed-set proteins (green triangles), which together with their interacting partners, may contain novel regulators of the EGFR signaling network. Proteins were extracted from the HPRD that interact directly with at least one of the seed set-proteins as well as a second-degree interactor (i.e. partner of the partners). A total of 533 proteins that satisfied these criteria were extracted from the database (blue and pink nodes), and a library of siRNAs targeting each candidate was constructed and subsequently used for the high-content Picchu-FLIM screen. Pink nodes represent proteins that were identified as hits from the siRNA screen, and therefore represent cases where protein knockdown resulted in a change in EGFR activity as measured by FRET-FLIM.

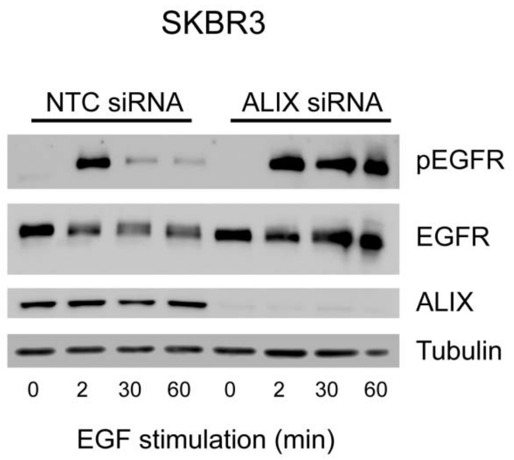

Figure S2. ALIX depletion results in sustained ligand-dependent EGFR phosphorylation in SKBR3 cells, Related to Figure 2.

Western blot of EGFR phosphorylation in NTC control and ALIX siRNA transfected SKBR3 human breast cancer cells stimulated with EGF for the indicated time points. Elevated and sustained EGFR phosphorylation is observed in ALIX knockdown cells, when compared with the NTC controls (Blot is one of two independent but similar results).

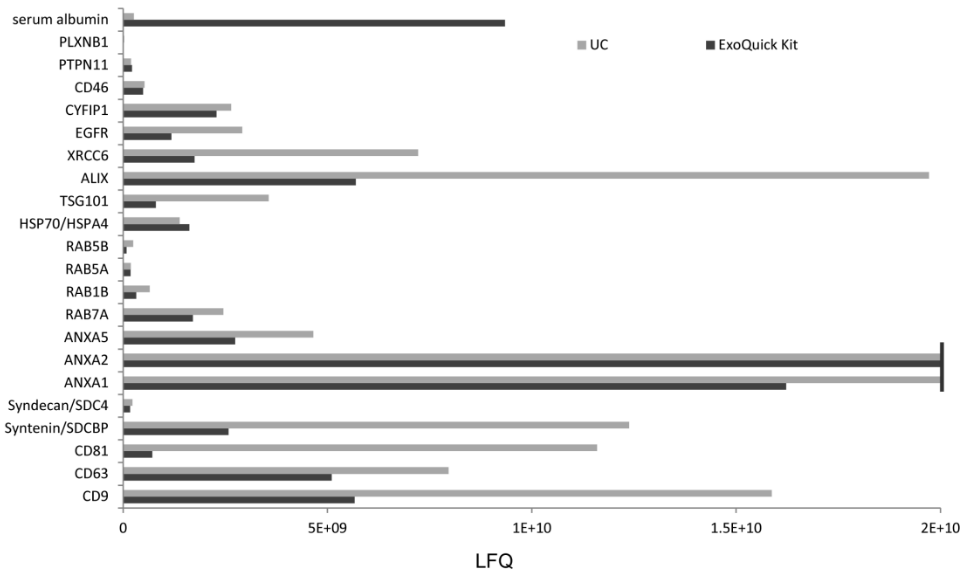

Figure S3. Comparison of proteomics data for exosome preparations obtained by ultracentrifugation and ExoQuick enrichment methods, Related to Figure 3.

Comparative proteomics analysis of HCC1954-derived exosomes isolated by ultracentrifugation (UC) or using the ExoQuick kit. Proteomics analysis reveals protein markers commonly associated with exosomes for both techniques used, although the ExioQuick method is associated with higher levels of the extracellular protein contaminant serum albumin. Exosomes were analyzed by LC-MS/MS. Relative label-free quantification (LFQ) intensities for indicated proteins are shown.

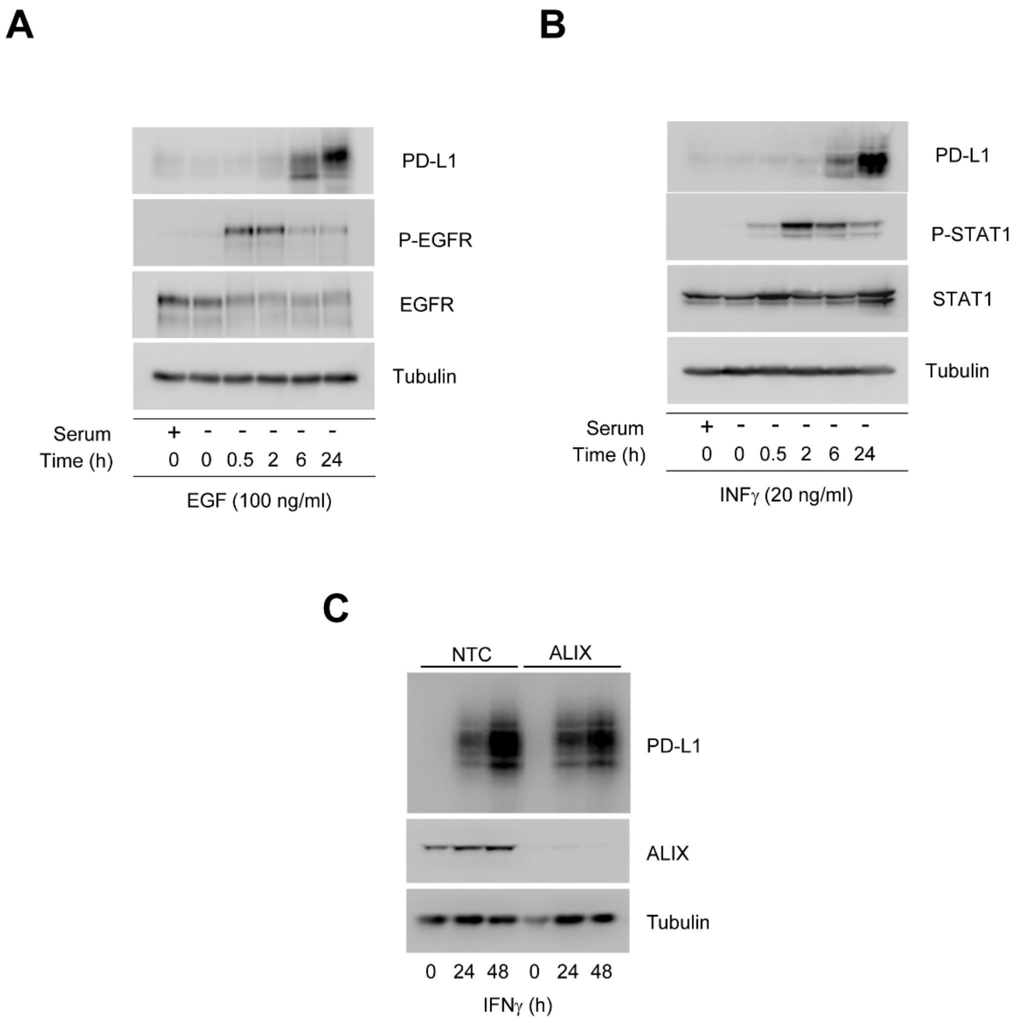

Figure S4. Ligand-dependent PD-L1 induction in HCC1954 cells, Related to Figure 4.

Representative western blots demonstrating the induction of PD-L1 protein expression in HCC1954 cells following their treatment with either EGF (A, 100 ng/ml) or IFNγ (B, 20 ng/ml). (C) Western blot showing the robust and prolonged induction of PD-L1 expression in both control and ALIX knockdown cells.

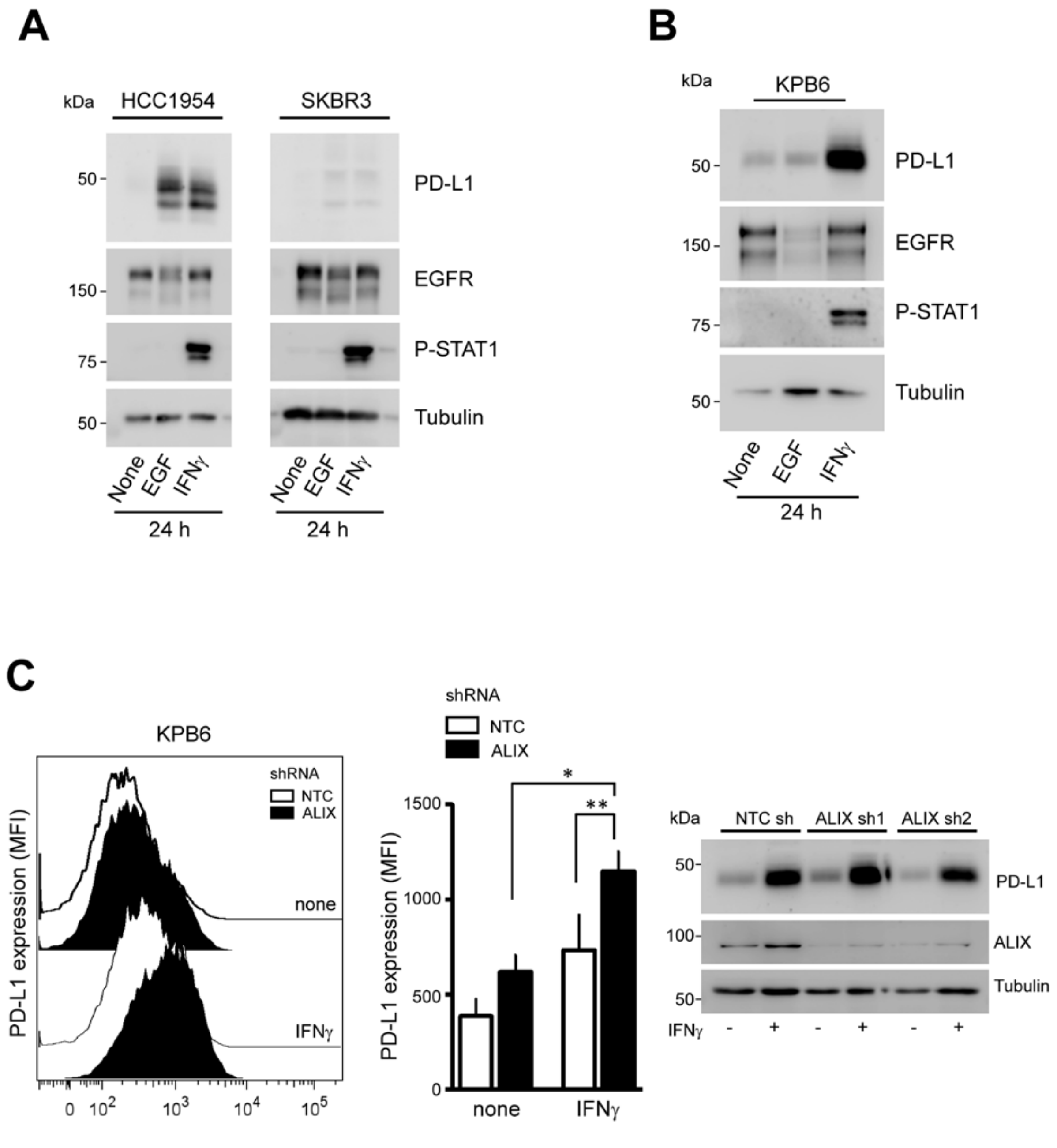

Figure S5. Ligand-dependent PD-L1 induction in various tumor cell lines, Related to Figure 5.

(A) Western blots comparing the effects of EGF and IFNγ stimulation on PD-L1 expression in HCC1954 (left column) and SKBR3 (right column) human breast carcinoma cells. Membranes for the two different cell lines were imaged side-by-side using the same exposure to enable the direct comparison of protein expression levels. (B) Western blots demonstrating the effects of EGF and IFNγ stimulation on PD-L1 expression in mouse KBP6 tumor cells. (C) Flow cytometry data (left panel) and corresponding statistical analysis (middle panel) of surface PD-L1 expression in NTC and ALIX shRNA KPB6 cells following 24 h stimulation with IFNγ. Data shown represent the pooled values of PD-L1 MFI for the two stable ALIX shRNA KPB6 cell lines used in three independent experiments. Western blot (right panel) demonstrating total PD-L1 and ALIX expression in NTC and ALIX shRNA KPB6 cells used in flow cytometry assays.

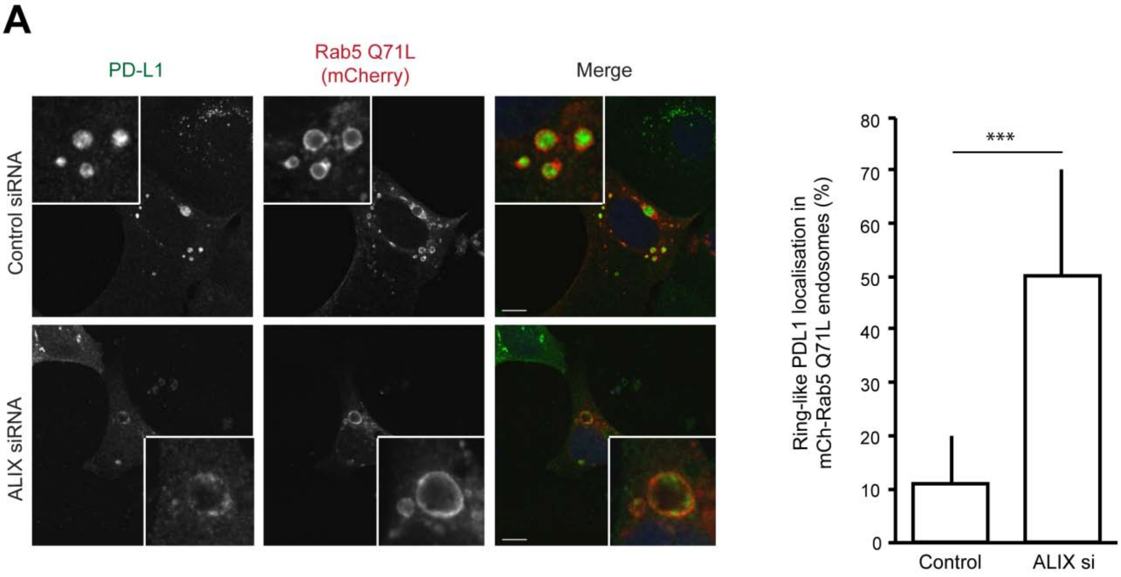

B

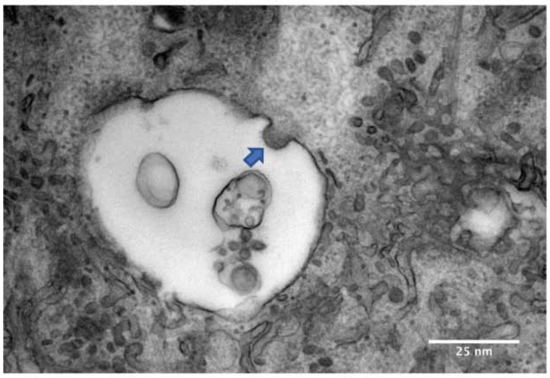

Budding profile	NTC sh	ALIX sh	
Yes	4	24	p<0.001
No	96	76	
	100	100	

Figure S6. ILV incorporation of PD-L1 is suppressed in ALIX knockdown cells, Related to Figure 5.

(A) Impaired incorporation of PD-L1 into the ILVs of ALIX knockdown cells. Confocal analysis (left-hand panel) and associated statistical analysis (right-hand bar graph) of PD-L1 localization in the MVBs of control and ALIX knockdown cells overexpressing the Rab5 Q71L mutant. IFNγ-stimulated control and ALIX siRNA cells were transfected with mCherry-tagged Rab5 Q71L plasmid and subsequently fixed and stained with anti-PD-L1 antibody. Rab5 Q71L overexpression results in the enlargement of endosomes, facilitating an analysis of PD-L1 staining within the lumen and at the limiting membrane of these structures. PD-L1 staining is largely exclude from the MVB lumen in ALIX knockdown cells and is instead confined to the limiting membrane (Control siRNA, n = 214 endosomes across 25 cells; ALIX siRNA, N = 125 endosomes across 25 cells. $P < 0.05$, two-tailed T-test). (B) Increased frequency in the observation of budding profiles in MVBs of ALIX knockdown cells. Example TEM image (left-hand panel) showing a budding profile (indicated by blue arrow) associated with an MVB of an ALIX knockdown cell. The bud, which is a precursor of an ILV, protrudes inwards towards the MVB lumen but remains attached to the limiting membrane. The presence of budding profiles was determined for 100 MVBs from cells across N = 4 independent experiments for each NTC and ALIX shRNA treatment group (right-hand table). Pooled scores were analyzed using a two-tailed Fisher's exact test.

Supplemental Tables

Category	Gene name	Gene ID	Characteristics of encoded protein
**Transmembrane proteins/receptors**	*EGFR*	1956	Transmembrane receptor tyrosine kinase
	PLXNB1	5364	Transmembrane semaphorin receptor, regulator of c-MET
	FASLG	356	Transmembrane receptor associated with apoptotic signaling
	CD46	4179	Type I transmembrane protein implicated in compliment signaling
	TMEM8B	51754	Transmembrane protein of unknown function
**Membrane receptor regulators/adaptors**	*SHC3*	53358	SH2-containing signaling adapter/docking protein
	NUMB	8650	Notch1 interacting protein/negative regulator of Notch signaling
	CBLC	23624	An E3 ubiquitin protein ligase
**Extracellular ligands**	*LIF*	3976	Pleotropic cytokine with a broad range of associated functions
**Regulators/effectors of Rho-family GTPases**	*DOCK1*	1793	Guanine nucleotide exchange factor
	MCF2	4168	Guanine nucleotide exchange factor
	TRIO	7204	Guanine nucleotide exchange factor
	CDC42EP1	11135	Effector of the Rho family GTPase Cdc42
	CYFIP1	23191	Component of the WAVE1 complex
**Non-receptor kinases**	*PRKAR1A*	5573	Regulatory subunit of type I cAMP-dependent protein kinase
**Non-receptor phosphatases**	*PTPN11*	5781	Protein tyrosine phosphatase
**Endosomal trafficking**	*PDCD6IP (ALIX)*	10015	ESCRT protein associated with EV biogenesis
**DNA binding/processing**	*XRCC6*	2547	Single-stranded DNA-dependent helicase
	ID2	3398	Inhibitor of DNA binding
**Other**	*C14orf1*	11161	Protein of unknown function

Table S1. Proteins identified as regulators of EGFR in the high-content RNAi screen, Related to Figure 2.

Table listing the characteristics and IDs of the 19 genes identified as regulators of EGFR from the high content siRNA screen. EGFR is also included for completeness because it was the positive control and identified as a hit protein in the biosensor high-content screen.

Gene name	Exosomal cargo	URL	No. of citations	ISEV score
EGFR	Yes	http://exocarta.org/gene_summary?gene_id=1956	11	32
PLXNB1	Yes	http://exocarta.org/gene_summary?gene_id=5364#211	1	4
FASLG	Yes	http://exocarta.org/gene_summary?gene_id=356	3	5
CD46	Yes	http://exocarta.org/gene_summary?gene_id=4179	7	26
TMEM8B	No			
SHC3	No			
NUMB	Yes	http://exocarta.org/gene_summary?gene_id=8650	1	4
CBLC	No			
LIF	No			
DOCK1	Yes	http://exocarta.org/gene_summary?gene_id=1793	2	8
MCF2	No			
TRIO	Yes	http://exocarta.org/gene_summary?gene_id=7204	1	3
CDC42EP1	Yes	http://exocarta.org/gene_summary?gene_id=511099	1	0
CYFIP1	Yes	http://exocarta.org/gene_summary?gene_id=23191	11	38
PRKAR1A	Yes	http://exocarta.org/gene_summary?gene_id=5573	4	13
PTPN11	Yes	http://exocarta.org/gene_summary?gene_id=5781	3	10
PDCD6IP (ALIX)	Yes	http://exocarta.org/gene_summary?gene_id=10015	36	106
XRCC6	Yes	http://exocarta.org/gene_summary?gene_id=2547	7	27
ID2	No			
C14orf1	Yes	http://exocarta.org/gene_summary?gene_id=11161	1	2

Table S2. Genes identified as regulators of EGFR are associated with the exosome cargo, Related to Figure 2.

Table listing genes identified as regulators of EGFR from the high content siRNA screen and their known association with the exosomal cargo: genes were classed as encoding exosomal proteins based on their presence in the ExoCarta database (http://exocarta.org/index.html). URLs for specific gene entries are provided in the third column. "No. of citations" refers to the number of independent studies recorded in ExoCarta for a given gene. Entries in the ExoCarta database are assessed against five criteria laid down by the ISEV (Keerthikumar et al., 2016). These criteria collectively provide an indication of how rigorous a study was in its characterization of extracellular vesicles, and therefore provides a qualitative measure of the confidence with which the vesicles studied can be considered to be *bona fide* exosomes. In the table above, the "ISEV score" is the sum of all fulfilled ISEV criteria from all annotated studies for a given gene in the ExoCarta database.

<table>
<tr><th rowspan="3">Gene name</th><th colspan="6">Protein expression</th></tr>
<tr><th rowspan="2">Normal human breast tissue</th><th colspan="5">Human breast cancer tissue</th></tr>
<tr><th>High</th><th>Medium</th><th>Low</th><th>None</th><th>Total</th></tr>
<tr><td>EGFR</td><td>None detected</td><td>0</td><td>2</td><td>1</td><td>7</td><td>10</td></tr>
<tr><td>PLXNB1</td><td>Medium</td><td>2</td><td>8</td><td>1</td><td>0</td><td>11</td></tr>
<tr><td>FASLG</td><td>None detected</td><td>0</td><td>0</td><td>0</td><td>11</td><td>11</td></tr>
<tr><td>CD46</td><td>High</td><td>0</td><td>3</td><td>7</td><td>2</td><td>12</td></tr>
<tr><td>NUMB</td><td>High</td><td>2</td><td>4</td><td>1</td><td>2</td><td>9</td></tr>
<tr><td>DOCK1</td><td>High</td><td>1</td><td>2</td><td>2</td><td>7</td><td>12</td></tr>
<tr><td>TRIO</td><td>Medium</td><td>0</td><td>4</td><td>6</td><td>1</td><td>11</td></tr>
<tr><td>CDC42EP1</td><td>Low</td><td>0</td><td>0</td><td>1</td><td>10</td><td>11</td></tr>
<tr><td>CYFIP1</td><td>Medium</td><td>0</td><td>0</td><td>9</td><td>2</td><td>11</td></tr>
<tr><td>PRKAR1A</td><td>Low</td><td>1</td><td>6</td><td>3</td><td>13</td><td>12</td></tr>
<tr><td>PTPN11</td><td>Medium</td><td>5</td><td>5</td><td>1</td><td>0</td><td>11</td></tr>
<tr><td>PDCD6IP (ALIX)</td><td>Medium</td><td>0</td><td>3</td><td>7</td><td>1</td><td>11</td></tr>
<tr><td>XRCC6</td><td>High</td><td>12</td><td>0</td><td>0</td><td>0</td><td>12</td></tr>
<tr><td>C14orf1</td><td>Low</td><td>0</td><td>1</td><td>3</td><td>7</td><td>11</td></tr>
</table>

Table S3. Expression profile of genes identified as regulators of EGFR in normal and cancerous breast tissues, Related to Figure 2.

Table listing genes identified as regulators of EGFR from the high content siRNA screen and their protein expression in normal human breast tissues and breast cancer tissues according to the Human Protein Atlas database (http://www.proteinatlas.org). The database contained immunohistochemical expression data for all 13 of the hit proteins associated with exosomes.

**Antibody target**	**Supplier**	**Code/clone**	**Assay**
EGFR	Cell Signaling Technology	#4267	**WB/DB**
p-EGFR	Cell Signaling Technology	#4407/53A5	**WB/DB**
CrkII	Cell Signaling Technology	#3492	**WB**
p- CrkII	Cell Signaling Technology	#3491	**WB**
STAT1	Cell Signaling Technology	#9172	**WB/DB**
p-STAT1	Cell Signaling Technology	#9167	**WB/DB**
ALIX	Cell Signaling Technology	#2171	**WB/DB**
Calnexin	Cell Signaling Technology	#2679	**DB**
PD-L1	Cell Signaling Technology	#13684	**WB/DB/IF/IHC**
CD63	GenTex	#GTX28219	**DB**
CD63	Developmental Studies Hybridoma Bank	#H5C6	**IF**
CD63	gift from Fedor Berditchevski	N/A	**IEM**
TSG101	GenTex	#GTX70255	**WB**
EpCAM	Sigma	#SAB4700423	**DB**
α-actin	Sigma	#A2668	**WB**
Tubulin	Sigma	#T6074	**WB**
CD45-AF700	BioLegend	#103127/30-F11	**FC**
CD3-PECy7	BioLegend	#100319/145-2C11	**FC**
CD4-BV785	BioLegend	#100453/GK1.5	**FC**
CD8-PerCpCy5.5	BioLegend	#100733/53-6.7	**FC**
CD25-PE	BioLegend	# 102007/PC61.5	**FC**
Granzyme B-APC	BioLegend	# 372203/QA16A02	**FC**
Foxp3-eFluor450	eBioscience	# 48-5773-82/FJK-16s	**FC**

Table S4. Table of antibodies used in this study, Related to Experimental Procedures.

The table summarizes the antibodies used in this study, including associated application(s) and product and supplier information. WB; western blotting, DB; dot-blotting, FC; flow cytometery, IF; immunofluorescence, IHC; immunohistochemistry, IEM; immuno-electron microscopy. Antibody suppliers listed include Cell Signaling Technology (Danvers, MA, USA), GenTex (Letchworth Garden City, UK), Sigma-Aldrich Company Ltd. (Dorset, England), BioLegend (London, UK), and eBioscience (San Diego, CA, USA).

Supplemental Experimental Procedures

Mass spectrometry analysis of exosomes.

Pelleted exosomes were lysed in 100 µL of 1% SDS buffer, sonicated and clarified by centrifugation. The protein concentration was determined by a BCA assay (Pierce). Approximately 40 µg of protein was processed according to the FASP protocol and reduced with 100 mM DTT for 5 min at 95 °C. SDS was removed by sequential washes using 30kDa cut-off spin columns (Sartorius). The sample was then alkylated with 55 mM iodoacetamide in 50 mM ABC for 1 h at room temperature and subsequently washed by sequential wash steps with 50 mM ABC. Then, samples were digested with trypsin (Promega) at 37°C over night. Peptides were recovered by centrifugation through the cut-off membrane. Peptide concentration was determined by 280 nM absorption. Peptides were acidified (4µg per sample) with 0.5% trifluoroacetic acid and desalted with homemade STAGE-tips (Wisniewski et al., 2009).

Tryptic peptides were separated on an Ultimate Ultra3000 chromatography system incorporating an auto sampler and analyzed using a Q-Exactive mass spectrometer (Thermo Scientific, Germany). The tryptic peptides (5 µL of each sample) were loaded on a homemade column (100 mm length, 75 µm inside diameter) packed with 1.9 µm ReprosilAQ C_{18} (Dr.Maisch, Germany) and separated by an increasing acetonitrile gradient, using a 60-min reverse-phase gradient (from 3–32 % Acetonitrile) at a flow rate of 250 nL/min, operated in positive ion mode with a capillary temperature of 320 °C, with a potential of 2300 V applied to the column. Data were acquired with the mass spectrometer operating in automatic data-dependent switching mode, selecting the 12 most intense ions prior to tandem MS (MS/MS) analysis. An ion selection limit of 8300 was applied for the counts, and selected ions were dynamically excluded for the next 40 s. Mass spectra were analyzed using the label-free quantitation MaxQuant Software package. All the samples were analyzed as two technical replicates and three biological triplicates.

Data was analyzed using the MaxQuant software package. Raw data files were searched against a human database (Uniprot HUMAN) with methionine oxidation as variable modification using a mass accuracy of 4.5 ppm and 0.01 false discovery rate (FDR) at both peptide and protein level to exclude false-positives. Each file was considered as separate in the experimental design; the replicates of each condition were grouped for the subsequent statistical analysis which was performed using the Andromeda software suite. Reverse and proteins identified solely with modified peptides were excluded. LFQ intensities were log(2) transformed. All entries were deleted if at least one sample group did not have all the values for all replicates. Results were cleaned for reverse and contaminants and a list of significant interactions was determined based on average ratio and ANOVA T-test using a protein level cut-off of 1.5- or 0.67-fold change.

Supplemental References

Fruhwirth, G.O., Fernandes, L.P., Weitsman, G., Patel, G., Kelleher, M., Lawler, K., Brock, A., Poland, S.P., Matthews, D.R., Keri, G.*, et al.* (2011). How Forster resonance energy transfer imaging improves the understanding of protein interaction networks in cancer biology. Chemphyschem : a European journal of chemical physics and physical chemistry *12*, 442-461.

Keerthikumar, S., Chisanga, D., Ariyaratne, D., Al Saffar, H., Anand, S., Zhao, K., Samuel, M., Pathan, M., Jois, M., Chilamkurti, N.*, et al.* (2016). ExoCarta: A Web-Based Compendium of Exosomal Cargo. J Mol Biol *428*, 688-692.

Weitsman, G., Lawler, K., Kelleher, M.T., Barrett, J.E., Barber, P.R., Shamil, E., Festy, F., Patel, G., Fruhwirth, G.O., Huang, L.*, et al.* (2014). Imaging tumour heterogeneity of the consequences of a

PKCalpha-substrate interaction in breast cancer patients. Biochemical Society transactions *42*, 1498-1505.
Wisniewski, J.R., Zougman, A., and Mann, M. (2009). Combination of FASP and StageTip-based fractionation allows in-depth analysis of the hippocampal membrane proteome. Journal of proteome research *8*, 5674-5678.
