# Supplementary material for: ALIX Regulates Tumor-Mediated Immunosuppression by Controlling EGFR Activity and PD-L1 Presentation
Source: Cell Rep. 2018 Jul 17;24(3):630–41. doi: 10.1016/j.celrep.2018.06.066 (PMC6077252; doi:10.1016/j.celrep.2018.06.066)
Supplement: Document S2. Article plus Supplemental Information [file mmc2.pdf]

Cell Reports

Article

ALIX Regulates Tumor-Mediated Immunosuppression by Controlling EGFR Activity and PD-L1 Presentation

Graphical Abstract

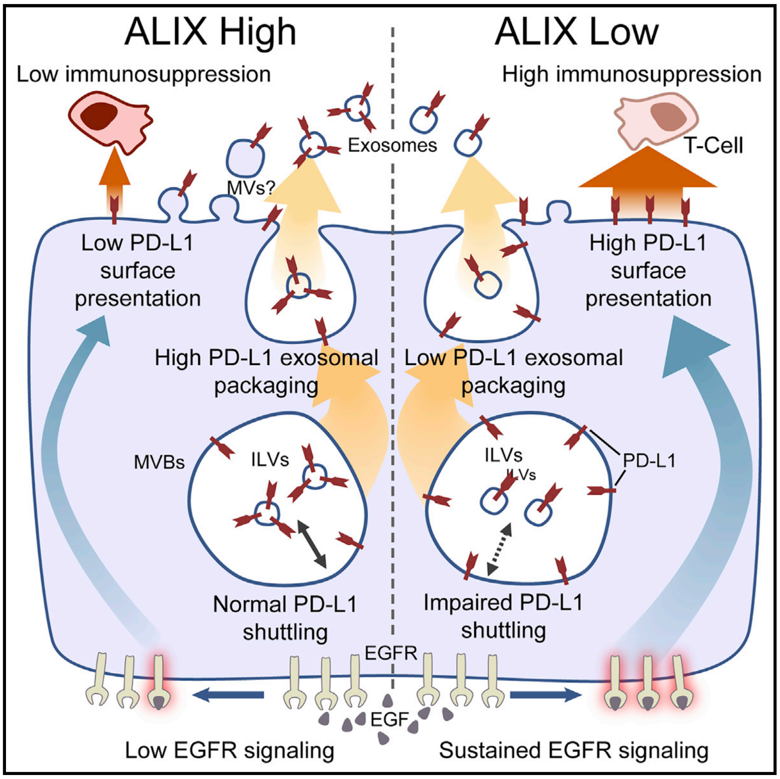

Highlights

- ALIX regulates EGFR activity and PD-L1 surface presentation in breast cancer cells
- PD-L1 is packaged into the exosomes of breast cancer cells
- ALIX depletion results in reduced exosomal and increased surface PD-L1 expression
- Increased surface PD-L1 is associated with an enhanced immunosuppressive phenotype

Authors

James Monypenny, Hanna Milewicz, Fabian Flores-Borja, ..., Borivoj Vojnovic, Jeremy G. Carlton, Tony Ng

Correspondence

jeremy.carlton@kcl.ac.uk (J.G.C.), tony.ng@kcl.ac.uk (T.N.)

In Brief

Monypenny et al. show that the ESCRT-related protein ALIX regulates two clinically important proteins in breast cancer; namely, EGFR, a receptor linked to cell survival, and PD-L1, an immune checkpoint protein. ALIX is, therefore, associated with pathways that drive both cell-autonomous and non-cell-autonomous mechanisms of tumor survival.

Monypenny et al., 2018, Cell Reports *24*, 630–641
July 17, 2018 © 2018 The Author(s).
https://doi.org/10.1016/j.celrep.2018.06.066

OPEN ACCESS
CellPress

Cell Reports
Article

ALIX Regulates Tumor-Mediated Immunosuppression by Controlling EGFR Activity and PD-L1 Presentation

James Monypenny,[1,17] Hanna Milewicz,[1,17] Fabian Flores-Borja,[1,2,17] Gregory Weitsman,[1,17] Anthony Cheung,[1,2] Ruhe Chowdhury,[1,13] Thomas Burgoyne,[15] Appitha Arulappu,[1] Katherine Lawler,[1,3] Paul R. Barber,[1,14] Jose M. Vicencio,[14] Melanie Keppler,[1] Wahyu Wulaningsih,[6] Sean M. Davidson,[5] Franca Fraternali,[7] Natalie Woodman,[8] Mark Turmaine,[9] Cheryl Gillett,[8] Dafne Franz,[14] Sergio A. Quezada,[14] Clare E. Futter,[15] Alex Von Kriegsheim,[10] Walter Kolch,[10,11,12] Borivoj Vojnovic,[4] Jeremy G. Carlton,[13,16,*] and Tony Ng[1,2,14,18,*]
[1]Richard Dimbleby Department of Cancer Research, Randall Division and Division of Cancer and Pharmaceutical Sciences, King's College London, Guy's Medical School Campus, London SE1 1UL, UK
[2]KCL Breast Cancer Now Research Unit, Department of Research Oncology, Guy's Hospital, King's College London, London SE1 9RT, UK
[3]Institute for Mathematical and Molecular Biomedicine, King's College London, Guy's Medical School Campus, London SE1 1UL, UK
[4]Department of Oncology, Cancer Research UK and Medical Research Council Oxford Institute for Radiation Oncology, University of Oxford, Oxford OX3 7DQ, UK
[5]Hatter Cardiovascular Institute, University College London, 67 Chenies Mews, London WC1E 6HX, UK
[6]Cancer Epidemiology Group, Division of Cancer Studies, King's College London, London, UK
[7]Bioinformatics and Computational Biology, Randall Division, King's College London, Guy's Medical School Campus, London SE1 1UL, UK
[8]KHP Cancer Biobank, King's College London, Innovation Hub, Guy's Cancer Centre, London SE1 9RT, UK
[9]Division of Biosciences, University College London, Gower Street, London WC1E 6BT, UK
[10]Systems Biology Ireland, University College Dublin, Belfield, Dublin 4, Ireland
[11]Conway Institute of Biomolecular and Biomedical Research, University College Dublin, Belfield, Dublin 4, Ireland
[12]School of Medicine, University College Dublin, Belfield, Dublin 4, Ireland
[13]Division of Cancer and Pharmaceutical Sciences, King's College London, Guy's Hospital, Great Maze Pond, London, UK
[14]UCL Cancer Institute, Paul O'Gorman Building, University College London, London WC1E 6DD, UK
[15]UCL Institute of Ophthalmology, 11-43 Bath Street, London EC1V 9EL, UK
[16]Organelle Dynamics Laboratory, The Francis Crick Institute, 1 Midland Road, London NW1 1AT, UK
[17]These authors contributed equally
[18]Lead Contact
*Correspondence: jeremy.carlton@kcl.ac.uk (J.G.C.), tony.ng@kcl.ac.uk (T.N.)
https://doi.org/10.1016/j.celrep.2018.06.066

SUMMARY

The immunosuppressive transmembrane protein PD-L1 was shown to traffic via the multivesicular body (MVB) and to be released on exosomes. A high-content siRNA screen identified the endosomal sorting complexes required for transport (ESCRT)-associated protein ALIX as a regulator of both EGFR activity and PD-L1 surface presentation in basal-like breast cancer (BLBC) cells. ALIX depletion results in prolonged and enhanced stimulation-induced EGFR activity as well as defective PD-L1 trafficking through the MVB, reduced exosomal secretion, and its redistribution to the cell surface. Increased surface PD-L1 expression confers an EGFR-dependent immunosuppressive phenotype on ALIX-depleted cells. An inverse association between ALIX and PD-L1 expression was observed in human breast cancer tissues, while an immunocompetent mouse model of breast cancer revealed that ALIX-deficient tumors are larger and show an increased immunosuppressive environment. Our data suggest that ALIX modulates immunosuppression through regulation of PD-L1 and EGFR and may, therefore, present a diagnostic and therapeutic target for BLBC.

INTRODUCTION

Epidermal growth factor receptor (EGFR) is either amplified or mutated in a variety of cancers and contributes significantly to tumorigenesis (Chong and Jänne, 2013; Ciardiello and Tortora, 2008). Basal-like breast cancer (BLBC), a subtype of disease with the worst therapeutic outcomes, commonly exhibits elevated EGFR expression (Burness et al., 2010; Irshad et al., 2011). While the subject of intense research, the complexity and plasticity of the tumor EGFR signaling network may underlie the poor response to current EGFR-targeted therapies (Hudis and Gianni, 2011). Thus, there is a need to seek additional molecular targets for improving EGFR targeting in basal-like disease.

Immune checkpoint blockade has introduced exciting possibilities to the field of targeted cancer therapy to inhibit tumor growth, with exploitation of programmed death-ligand 1 (PD-L1) showing great therapeutic promise (Pardoll, 2012). The recent demonstration of a direct association between activating EGFR mutations and increased PD-L1 expression in

 © 2018 The Author(s).
This is an open access article under the CC BY license (http://creativecommons.org/licenses/by/4.0/).

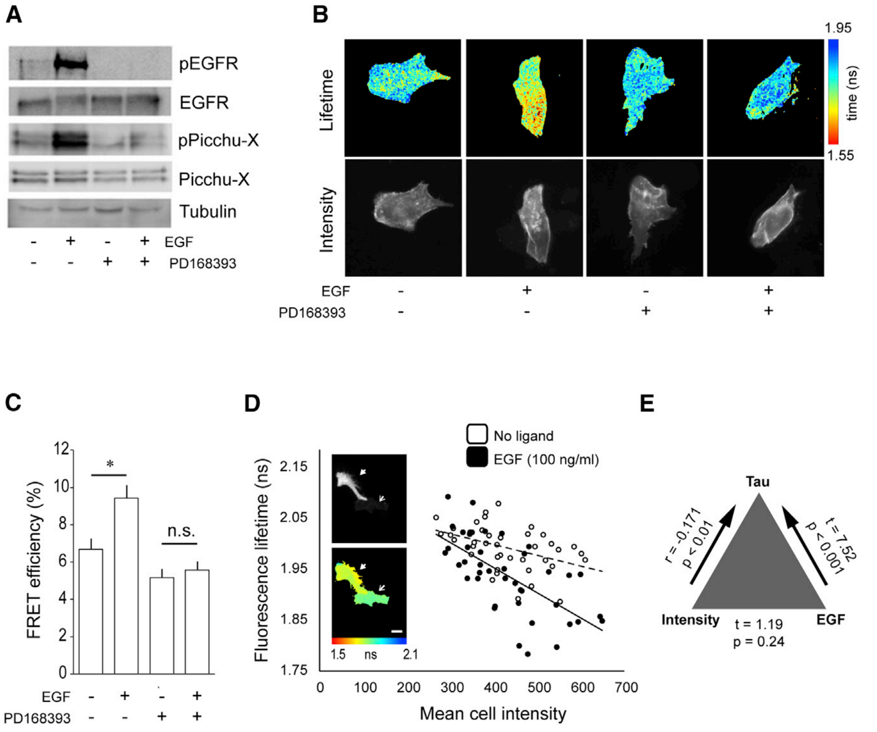

Figure 1. Biochemical Validation of the Picchu-X EGFR Activity Biosensor

(A) Lysates of cells transiently expressing the Picchu-X biosensor were examined by western blotting with the indicated antibodies.

(B) Donor fluorescence lifetime and intensity images for representative Picchu-X-expressing cells subjected to the indicated treatments. A PD16838-sensitive decrease in donor fluorescence lifetime is observed following treatment with EGF.

(C) Quantification of average FRET efficiencies for treatment groups shown in (B). Data are mean FRET efficiencies, and error bars represent SEM. n $\geq$ 12 for each group; significance determined using Student's t test (*$p < 0.05$). n.s., not significant.

(D) Population distribution of mean cell fluorescence lifetimes versus mean fluorescence intensities for Picchu-X-expressing cells treated as indicated. Donor fluorescence intensities (top) and associated lifetime maps (bottom) are indicated for two example cells with high (solid arrow) and low (empty arrow) levels of Picchu-X biosensor expression. Scale bar, 40 μm.

(E) Three-way correlation analysis of biosensor donor lifetime, biosensor expression, and ligand treatment. Data related to lifetime, biosensor expression, and ligand treatment for all cells included in the siRNA library screen were included in the correlation analysis. Two-tailed Pearson correlation coefficient (r) reported a statistically significant association between intensity and τ ($p < 0.01$). The difference in τ between treatment groups (t = t-statistic value) was highly significant ($p < 0.001$) and incorporates the significant association between intensity and τ. There was no significant association between intensity and treatment ($p = 0.24$).

non-small-cell lung cancer has shown an interdependence between cancer cell-autonomous (EGFR-dependent cell survival) and non-autonomous (PD-L1-dependent evasion of immune surveillance) mechanisms of tumor survival (Akbay et al., 2013; Murillo et al., 2014). Consequently, tumors harboring activating EGFR mutations benefit from two distinct but linked survival pathways, and targeting of mutant EGFR can impair the immunosuppressive phenotype of these tumors (Chen et al., 2015). However, targeting of wild-type receptors in human lung cancer cell lines has little effect on PD-L1 expression (Azuma et al., 2014), suggesting that the link between EGFR and PD-L1 expression is dependent on the kinase activity of the receptor (Akbay et al., 2013; Azuma et al., 2014). In BLBC, PD-L1 expression is frequently observed alongside elevated wild-type EGFR expression (Irshad et al., 2011; Nielsen et al., 2004), suggesting that the immunosuppressive characteristics of these tumors may be refractory to the effects of therapies targeting EGFR's kinase activity. A greater understanding of mechanisms driving EGFR signaling in breast cancer is, therefore, warranted; in particular, how EGFR activity in this disease affects immunosuppressive pathways mediated by PD-L1.

Here, we performed a bioinformatics-led RNAi screen in BLBC cells to identify regulators of EGFR activity. Among proteins identified were exosomal cargo proteins and proteins implicated in exosome biogenesis. We found that cells lacking the endosomal sorting complexes required for transport (ESCRT) component ALIX, a critical mediator of exosome biogenesis (Bissig and Gruenberg, 2014; Carlton, 2010), displayed enhanced EGFR activation, suggesting unexpected parallels between mechanisms of exosome biogenesis and regulation of EGFR activity. We found that PD-L1 is secreted on exosomes in an ALIX-dependent manner, and impaired exosomal release conferred an enhanced immunosuppressive phenotype on tumor cells that was dependent upon EGFR kinase activity. Our data suggest that downregulation of ALIX provides a mechanism for enhancing both EGFR activity and PD-L1-mediated evasion of anti-tumor immunity in BLBC, driving cell-autonomous and non-cell-autonomous mechanisms of tumor survival.

RESULTS

EGFR Activity Monitoring *In Situ* Using a FRET Biosensor

Fluorescence resonance energy transfer (FRET)-based biosensors enable *in situ* monitoring of signaling pathway activities in cells (Komatsu et al., 2011). The Picchu-X FRET biosensor (Kurokawa et al., 2001) is based on the CrkII adaptor protein, a direct target of EGFR phosphorylation (Hashimoto et al., 1998). Here, we use a modified version of Picchu-X that has been optimized for fluorescence lifetime imaging (FLIM) to monitor EGFR activity in HCC1954 BLBC cells.

Background biosensor activity in HCC1954 cells was low, indicating a minimal basal EGFR activity (Figure 1A). We observed a stimulation-dependent and PD168393-sensitive enhancement of Picchu-X phosphorylation (Figure 1A) and FRET/FLIM (Figures 1B and 1C), demonstrating the biosensor's specificity as a readout of EGFR activity. The relationship between mean donor lifetime and mean fluorescence intensity among untreated and EGF-treated populations was incorporated into the analysis to

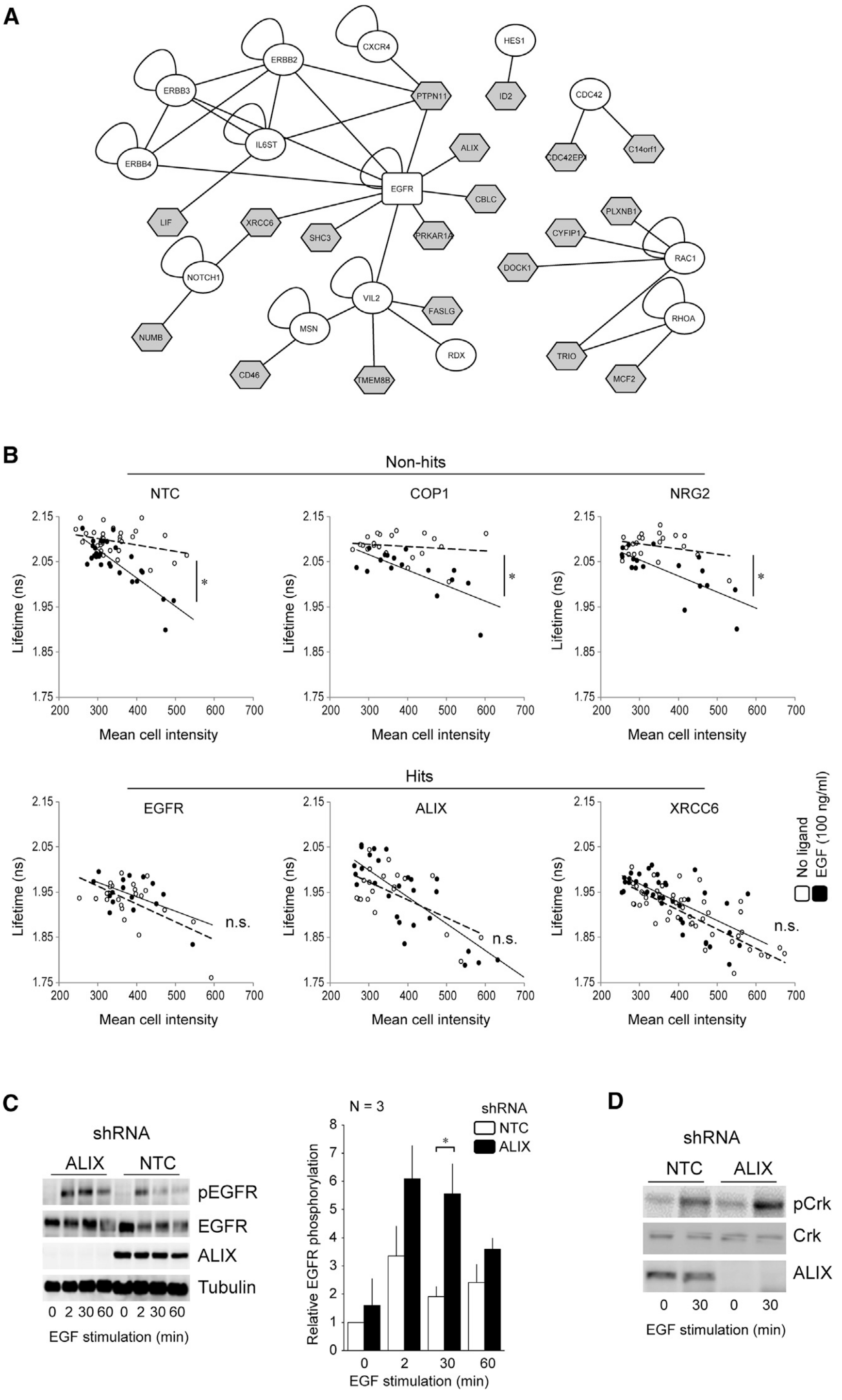

Figure 2. Picchu-X Screen to Identify Hits Affecting EGFR Activity

(A) The protein interaction sub-network of the 20 hits identified in a high-content siRNA screen and their connection to seed set proteins. The sub-network was extracted from a larger network of 533 proteins obtained from the Human Protein Reference Database (HPRD) based on their direct associations with seed-set proteins and 1 (or more) other network member (central rectangle indicates primary seed or EGFR; white ellipses indicate additional seeds; shaded polygons indicate screen hits).

(B) FLIM-intensity scatterplots and associated regression analysis of six example experimental groups (three non-hits and three hits). Each data point shows mean fluorescence lifetime plotted against mean fluorescence intensity for a single cell. Pearson correlation coefficients describe the association between lifetime and intensity for the EGF-treated (solid circles) and non-treated (empty circles) subgroups, and the difference in r values was used to evaluate the effects of target protein KD on EGFR activity (*$p < 0.05$).

(C and D) Lysates of the indicated EGF-stimulated HCC1954 cell lines were examined by western blotting. Bar chart in (C) summarizes densitometry analysis of phospho-EGFR levels normalized to tubulin ($n = 3$ independent experiments ± SEM (*$p < 0.05$, two-tailed Student's t test). Similarly, immunoblots for phospho-CrkII are shown in (D).

See also Figures S1 and S2.

account for lifetime variations arising from differences in biosensor expression levels (Figure 1D). The analysis confirmed that the association between EGF treatment and lifetime was unaffected by the level of biosensor expression (Figure 1E). This stringent method of analysis was applied to all lifetime data acquired from the high-content small interfering RNA (siRNA) screen.

Identification of Regulators of EGFR Using a FLIM-Based High-Content Screen

We determined the effects of the targeted knockdown of 533 candidate proteins on EGFR signaling *in situ* to identify regulators of EGFR among a network of candidate proteins extracted from a bioinformatics-led analysis of protein-interaction databases (Figure S1). Twenty hits were identified that abrogated the biosensor response to ligand (Figure 2A, gray nodes; Figure 2B, cf. difference in the slopes of linear regression lines for EGF versus non-EGF treatment cell populations within the example "non-hit" and "hit" siRNA experimental groups; Table S1). Details of the 20 hits identified in the screen are provided in Table S1, and their known interactions with members of the EGFR subnetwork are illustrated in Figure S1 (pink nodes).

ALIX Is a Negative Regulator of EGFR Activity

Of the 20 proteins identified in our screen, 14 were identified as exosomal cargo proteins (Table S2), suggesting that changes in exosomal sorting can influence EGFR activation. There is

increasing realization that cancer cells can exploit the exosomal pathway as a mechanism for driving tumor survival and dissemination (Bissig and Gruenberg, 2014; Ghossoub et al., 2014). Consequently, we sought to identify proteins providing an interface between oncogenic EGFR and exosomal components in BLBC. The expression of 12 of these 14 exosomal-related proteins has been confirmed in normal and cancerous human breast tissues (Table S3). Of these, the adaptor protein ALIX was selected for further investigation, given its association with the exosomal cargo and intraluminal vesicle (ILV) biogenesis (Table S2).

Using HCC1954 cells stably expressing ALIX short hairpin RNA (shRNA), we confirmed that stimulation-induced EGFR phosphorylation was both enhanced and prolonged in ALIX knockdown (KD) cells, when compared with non-targeting controls (NTCs; Figure 2C). Elevated levels of downstream signaling components (endogenous CrkII phosphorylation) were also observed in these cells following EGF stimulation (Figure 2D). Results were confirmed in SKBR3 breast cancer cells (Figure S2), demonstrating that this phenotype was not unique to HCC1954 cells.

ALIX Controls Exosomal Cargo Incorporation

ALIX is implicated in the multivesicular body (MVB) sorting pathway of ubiquitinated membrane receptors. This pathway is responsible for cargo incorporation into ILVs, linking ALIX directly to cargo sorting and exosome biogenesis (Baietti et al., 2012). Therefore, in addition to altering EGFR activity, ALIX KD may also affect the quality and composition of exosomes, with downstream consequences for tumor biology. Although procedures do not yet exist for the absolute purification of exosomes, sequential ultracentrifugation provides an established technique for the enrichment of extracellular vesicle (EV) subpopulations highly enriched with exosomes. Nanoparticle tracking analysis (NTA) revealed that ultracentrifugation-enriched particles from HCC1954 culture supernatants had a mean modal diameter of 86 nm, which lies within the range of 40–120 nm associated with exosomes (Figure 3A). Examination of these particles by transmission electron microscopy (TEM) revealed cup-shaped morphologies characteristic of exosomes, while immunogold labeling confirmed that they were positive for the exosome marker CD63 (Figure 3B). Western blotting confirmed the presence of the exosomal marker proteins TSG101, CD9, and ALIX (Figure 3C). Immunoblotting analysis revealed the enrichment and loss of positive and negative exosomal markers, respectively, in these ultracentrifugations. A visual analysis of extracellular vesicle preparations from HCC1954 cells stably expressing GFP-CD63 revealed a uniform field of fluorescent particles (Figure 3C), and a mass-spectrometry-based comparison of sequential ultracentrifugation and ExoQuick methods demonstrated that the centrifugation method was associated with a lower amount of extracellular protein contamination (Figure S3). Together, these data show that ultracentrifugation provides an effective method for the enrichment of exosomes from HCC1954 culture supernatants.

To test whether ALIX depletion alters the exosomal proteome of HCC1954 cells, exosomes from the culture supernatants of control and ALIX KD cells were examined by quantitative mass spectrometry. Liquid chromatography-tandem mass spectrometry (LC-MS/MS) identified a total of 3,700 proteins in the exosomes of parental HCC1954 cells. These included the core exosomal marker proteins CD63, CD9, and CD81 and proteins known to be associated with exosome biogenesis and endosomal trafficking, such as ALIX, syntenin, syndecan, and Rab5 (Figure S3).

Analysis of the exosomal proteome of ALIX KD cells revealed that 12 proteins were significantly downregulated and four were significantly upregulated, when compared with controls (Figure 3D). As expected, ALIX and syntenin (a cytoplasmic adaptor protein known to interact with ALIX as part of the exosome biogenesis machinery; Baietti et al., 2012) levels were significantly diminished in exosomes derived from ALIX KD cells.

PD-L1 Is Mis-secreted in the Absence of ALIX

We were intrigued to find that PD-L1, a protein of high clinical relevance because of its role in mediating tumor-associated immunosuppression, was significantly depleted from the exosomes of ALIX-suppressed cells. Basal PD-L1 expression in HCC1954 cells is elevated following cell stimulation with EGF or interferon (IFN)γ, the archetypal signaling factor associated with PD-L1 expression (Figures S4A and S4B). Exosomal PD-L1 incorporation correlates with cellular expression levels (Figure 4A), and we discovered that, while IFNγ treatment resulted in the robust induction and sustained expression of PD-L1 in both NTC and ALIX KD cells (Figure S4C), PD-L1 incorporation into exosomes was significantly reduced in ALIX KD cells (Figure 4B). Flow cytometry analysis of cell-surface PD-L1 expression in unstimulated and IFNγ-stimulated cells revealed that surface levels were elevated in ALIX KD cells (Figure 5A). These data suggest that exosomal release of PD-L1 occurs at the expense of surface PD-L1 levels and that ALIX controls the balance of receptor distribution between these membranes.

Because basal levels of PD-L1 are almost undetectable in SKBR3 cells, and are unchanged following EGF or IFNγ treatment (Figure S5A), we used mouse KPB6 tumor cells, which show a robust PD-L1 response to IFNγ (Figure S5B), as an alternative model to confirm the PD-L1 surface expression phenotype. Elevated surface PD-L1 levels were confirmed in ALIX-depleted and IFNγ-stimulated KPB6 cells (Figure S5C).

ALIX Regulates PD-L1 Sorting onto ILVs

To gain insight into the mechanism underlying the reduced exosomal packaging and enhanced cell-surface expression of PD-L1 in ALIX KD cells, we examined the subcellular localization of PD-L1. Immunocytochemistry revealed that, in addition to its cell-surface localization, PD-L1 also localized to both the limiting membrane and ILVs of CD63-positive MVBs in IFNγ-treated HCC1954 cells (Figure 5B). This was in agreement with our detection of PD-L1 in the exosomes of IFNγ-treated cells, because ILVs are the intracellular precursors of these extracellular vesicles. In ALIX KD cells, a greater proportion of PD-L1 was found at the limiting membrane of MVBs, rather than within the endosomal lumen (Figure 5B). Because MVBs are small subcellular structures, the presence of discrete intraluminal cargo is difficult to discern by epifluorescence microscopy. We used Rab5 Q71L expression to promote endosomal enlargement, facilitating the distinction between limiting membrane and endosomal lumen, which confirmed the limiting membrane

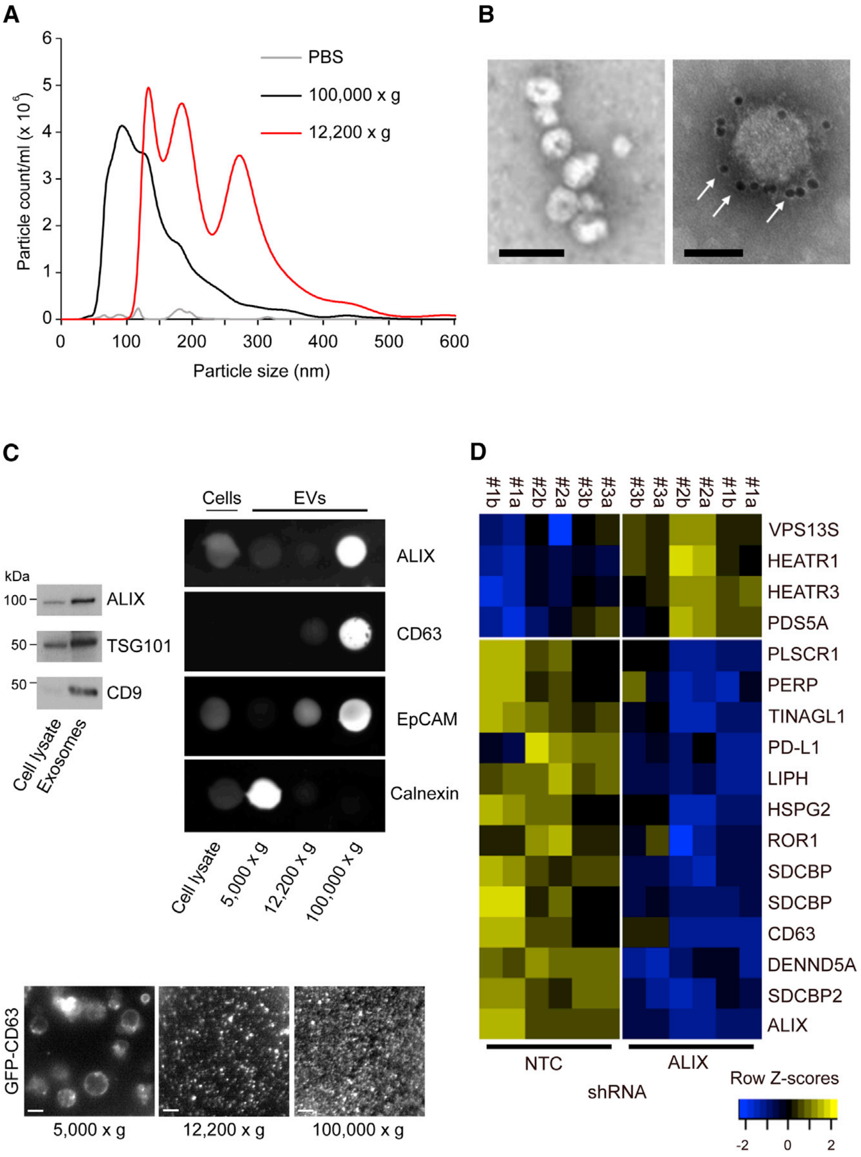

Figure 3. ALIX Regulates Exosomal Cargo Incorporation

(A) Summary of nanoparticle tracking analysis (NTA) showing the size distribution of EVs isolated from HCC1954 culture supernatants by ultracentrifugation. Each trace is representative of individual NTA performed on five samples. Bar graph shows mean modal size of exosomal and MV fractions from n = 5 traces ± SD (***p < 0.0001, two-tailed Student's t test).

(B) TEM of exosomes; arrows indicate CD63 immunolabeling. Scale bars, 100 nm (left image) and 50 nm (right image).

(C) Western blot demonstrating the presence of exosomal markers ALIX, TSG101, and CD9 in HCC1954 cell lysates and exosomes (equal protein loaded per lane) and showing the enrichment of these three exosomal markers in the corresponding exosome lanes. Dot-blots demonstrate the relative levels of positive and negative EV markers in the lysates of cells (5 μg per spot) and EVs (1 μg per spot) harvested after the indicated centrifugation steps. Epifluorescence images of GFP-CD63-labeled EVs harvested after the indicated centrifugation steps are shown.

(D) Heatmaps of changes in the exosomal proteome following ALIX KD in HCC1954 cells. Heatmaps are of protein expression across control (NTC) and ALIX KD (ALIX) samples and are displayed as row *Z* scores (each row is standardized by subtracting the mean value and dividing by the SD). Rows are ordered by mean fold change between NTC and ALIX sample groups (n = 3 independent experiments; proteins with mean fold change < 2 were excluded). For each treatment group (NTC or ALIX KD), the column number denotes an independent biological experiment, while the letter denotes a technical repeat. Gene name repeats indicate alternative splice variants.

See also Figure S3.

accumulation and luminal exclusion of PD-L1 in ALIX KD cells (Figure S6A). Interestingly, TEM analysis of MVBs from NTC and ALIX KD cells revealed that budding profiles (luminal invaginations of the limiting membrane of MVBs that are the precursors to ILVs) were more frequently observed in ALIX KD cells, when compared with controls (Figure S6B). When taken together with the immunofluorescence data, these findings suggest that there may be a defect in intra-endosomal budding associated with ALIX depletion. These data suggest that ALIX is required for incorporation of PD-L1 from the limiting membrane into ILVs, which would account for the diminished levels of exosomal PD-L1 and enhanced surface PD-L1 in ALIX-depleted cells.

ALIX Regulates the Immunosuppressive Properties of BLBC Cells by Regulating Surface PD-L1 Expression

The observation that loss of ALIX expression is associated with reduced exosomal packaging of PD-L1 and elevated cell-surface expression raises the intriguing possibility that cells with reduced ALIX expression exhibit enhanced immunosuppressive properties. To test this hypothesis, NTC and ALIX KD cells were either untreated or stimulated with IFNγ for 24 hr and co-cultured with activated primary human peripheral blood $CD3^{+}$ T lymphocytes to observe the effects of co-culture on lymphocyte proliferation. Flow cytometry analysis of T cells revealed an impairment in the proliferation of T cells co-cultured with ALIX-depleted tumor cells (Figure 5C). These findings demonstrate that ALIX depletion confers enhanced immunosuppressive properties on cancer cells.

Given that PD-L1 expression increases following EGFR or IFNγ receptor activation, and given that ALIX both regulates EGFR activity and modulates the exosomal and cell-surface distribution of PD-L1, we sought to determine whether EGFR signaling integrated with the IFNγ:PD-L1 signaling axis by examining whether the expression and cellular distribution of PD-L1 induced by IFNγ treatment in NTC and ALIX KD cells

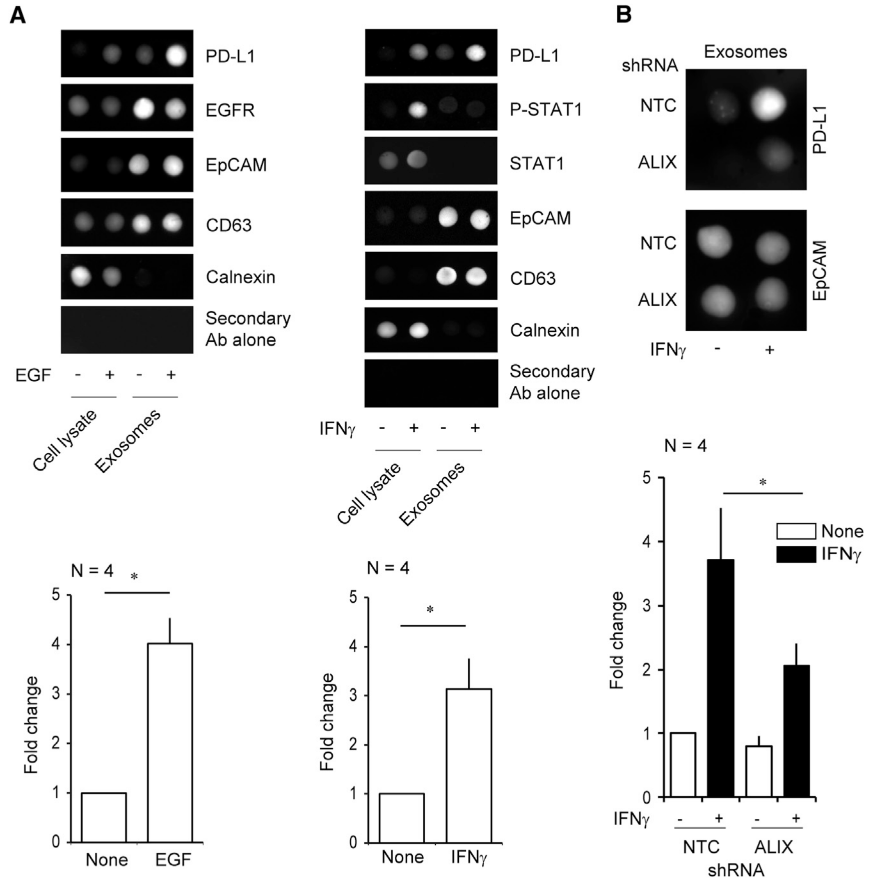

Figure 4. Ligand-Induced Expression and Exosomal Packaging of PD-L1 in HCC1954 Breast Cancer Cells

(A and B) Dot-blots (1 μg per spot) and associated densitometry analysis showing the EGF- and/or IFNγ-dependent induction of PD-L1 protein expression and exosomal packaging in HCC1954 cells (mean fold change ± SEM; *p < 0.05, two-tailed Student's t test) (A). (B) IFNγ-dependent induction of PD-L1 protein expression and exosomal packaging in NTC or ALIX KD equivalent cell lines. See also Figure S4.

was dependent upon EGFR. Treatment of ALIX KD cells with the EGFR inhibitor PD168393 prior to IFNγ stimulation both diminished the surface accumulation of PD-L1 (Figure 5A) and reduced the immunosuppressive effect of these cells in subsequent co-culture assays (Figure 5C). These data indicate that EGFR signaling contributes to the IFNγ- and PD-L1-dependent immunosuppressive response and suggest that ALIX integrates the signaling of two important regulators of tumor-mediated immunosuppression, modulating PD-L1 surface expression and the EGFR signaling required for the associated immunosuppressive phenotype.

ALIX and PD-L1 Expression Levels Are Inversely Associated in Human Breast Cancer

Our *in vitro* data suggest that modulation of ALIX expression provides a mechanism for regulating surface PD-L1 expression. Furthermore, we show that differences in ALIX expression translate into differences in immunomodulatory potency. To examine the relevance of this ALIX:PD-L1 signaling axis in human disease, we performed a tissue microarray analysis of PD-L1 protein expression on 189 tumor samples across different human breast cancer subtypes and combined this with ALIX gene expression analysis using mRNA isolated from matched tissue samples. Our analysis revealed a statistically significant inverse correlation between ALIX mRNA and PD-L1 protein expression in tumor cells (Figures 6A and 6B).

ALIX Suppresses Tumor Growth and the Immunosuppressive Microenvironment *In Vivo*

We next wanted to evaluate the impact of ALIX loss on the anti-tumor immune response *in vivo*. For this, we used the well-characterized and established syngeneic BALB/c mouse model with the 67NR breast cancer cell line, which demonstrates robust induction of PD-L1 in response to IFNγ (Figure 6C).

Ex vivo analysis of the relationship between lymphocytic invasion and the ALIX:PD-L1 expression ratio in parental 67NR tumors grown in the mammary fat pad of BALB/c female mice revealed a complex intratumoral relationship in which distinct regions of low ALIX:PD-L1 and high ALIX:PD-L1 expression ratio were associated with different CD3+T cell counts (Figure 6C). Low-ALIX:PD-L1 ratio pixels were less infiltrated by CD3+ T cells than the high-ALIX:PD-L1 ratio pixels within the same tumor (Figures 6C and 6D).

To determine the effects of ALIX depletion on tumor growth and immunosuppression, we generated 67NR cell lines stably expressing control and ALIX shRNAs and used them to induce orthotopic subcutaneous tumors in mice. These *in vivo* studies showed that ALIX loss resulted in significantly enhanced tumor volume at day 14 (time of animal culling), when compared with control tumor-bearing mice (Figure 7A). Furthermore, an increased frequency of CD4+ regulatory T cells (Figure 7B) and a decreased frequency of Granzyme B-expressing, tumor-infiltrating CD4+ and CD8+ T cells (Figure 7C) were observed at day 14. Together, these results suggest that reduced ALIX expression contributes to the establishment of an immunosuppressive tumor microenvironment, leading to more aggressive tumor growth.

These observations suggest that ALIX is a regulator of both the surface expression and immunomodulatory potency of PD-L1 in breast cancer, in part, through its regulation of EGFR.

DISCUSSION

EGFR is abnormally and heterogeneously expressed in breast cancers (Nuciforo et al., 2015), and identifying factors that can

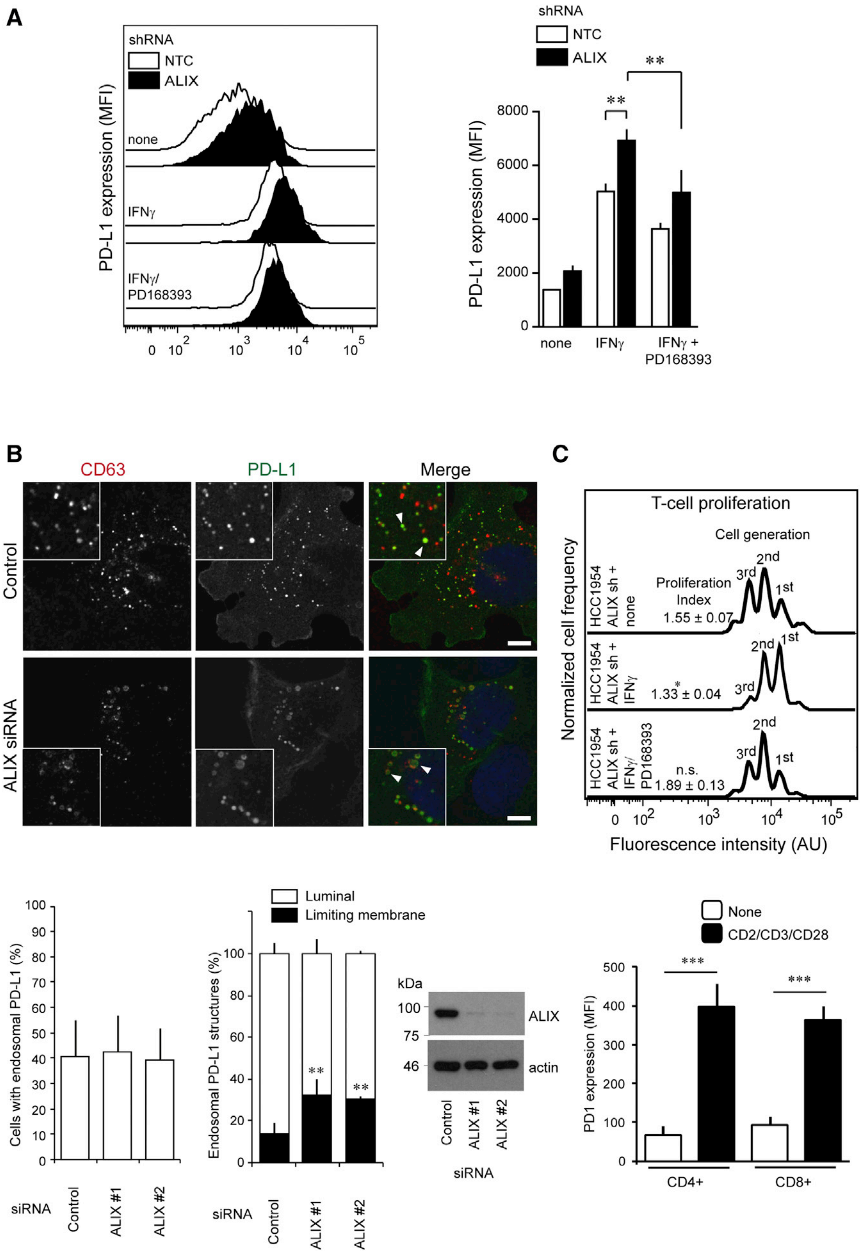

Figure 5. ALIX Regulates the Immunosuppressive Properties of HCC1954 Cells via a Redistribution of PD-L1

(A) Flow cytometry data and statistical analysis of surface PD-L1 expression in NTC and ALIX KD HCC1954 cells. Bars represent mean fluorescence intensities ± SEM (**$p < 0.01$, ANOVA).

(B) Top: HCC1954 cells were transfected with indicated siRNAs, treated with IFNγ, fixed, stained using antibodies against endogenous CD63 and PD-L1, and counterstained with DAPI. The percentage of cells displaying endosomal PD-L1 was calculated (bottom left graph, mean ± SD), and the percentages of CD63-positive PD-L1 decorated structures were scored: control, 63 ± 4.2%; ALIX #1, 64 ± 4.7%, n.s. (not significant); ALIX #2, 59 ± 2.7%, n.s. 100 cells per experiment, n = 4. Intraendosomal PD-L1 localization (right graph), was also scored; 3,977 endosomes from 10 fields of view per experiment were scored over 4 independent experiments (mean ± SD, **$p < 0.01$, two-tailed Student's t test). Lysates were examined by western blotting. Arrowheads highlight luminal and limiting membrane accumulation of PD-L1 in MVBs of control and ALIX KD cells, respectively. Scale bars, 10 μm.

(C) Cell-tracker dye intensity histograms of CD8+ T cell proliferation from labeled T cell-tumor cell co-culture assays. Cell proliferation indices are shown for each treatment group with their associated SEM (*$p < 0.05$ versus control group; n: 5 independent experiments for each treatment group). Bar chart summarizes the flow cytometry data of surface PD-1 expression in naive and CD2-, CD3-, and CD28-stimulated CD4+ and CD8+ T cell subpopulations (***$p < 0.001$, Student's t test; data are from n = 5 independent experiments).

See also Figures S5 and S6.

delineate patient subsets for more effective therapies is of significant clinical interest (Carey et al., 2012; Masuda et al., 2012). EGFR and immune checkpoint combination strategies are currently being tested in clinical trials (Ahn et al., 2016), and while PD-L1/PD1 pathway blockade is a promising therapeutic approach in oncology, our understanding of PD-L1 regulation remains incomplete. Future success of combination therapies will depend on better understanding of growth factor:PD-L1 signal crosstalk.

We have shown that ALIX is a negative regulator of EGFR activity, that its depletion significantly augments IFNγ-induced PD-L1 surface expression in human breast cancer cells, and that this upregulation is EGFR activity dependent. We have, therefore, extended the receptor tyrosine kinase (RTK) and immune checkpoint molecular interdependence phenomenon, previously only observed in EGFR mutated lung cancers (Akbay et al., 2013), to BLBC, where PD-L1 expression is frequently observed and where elevated EGFR expression is associated with poor survival.

PD-L1 has been identified in exosomes isolated from urine or plasma (Moon et al., 2011; Sabapatha et al., 2006) but has not previously been detected in the exosomes from cancer cells. Using exosomal proteome profiling, we found that HCC1954 cells package PD-L1 into exosomes and that ALIX depletion impairs this exosomal incorporation. Correlating with impaired ILV and exosomal incorporation of PD-L1, ALIX-depleted cells exhibit enhanced surface levels of PD-L1, conferring an enhanced immunosuppressive phenotype on these cells. The change in partitioning of PD-L1 between the exosomal and cell-surface compartments was associated with an altered pattern of PD-L1 localization at the endosome; namely, a loss of luminal PD-L1 and an accumulation of this protein at the

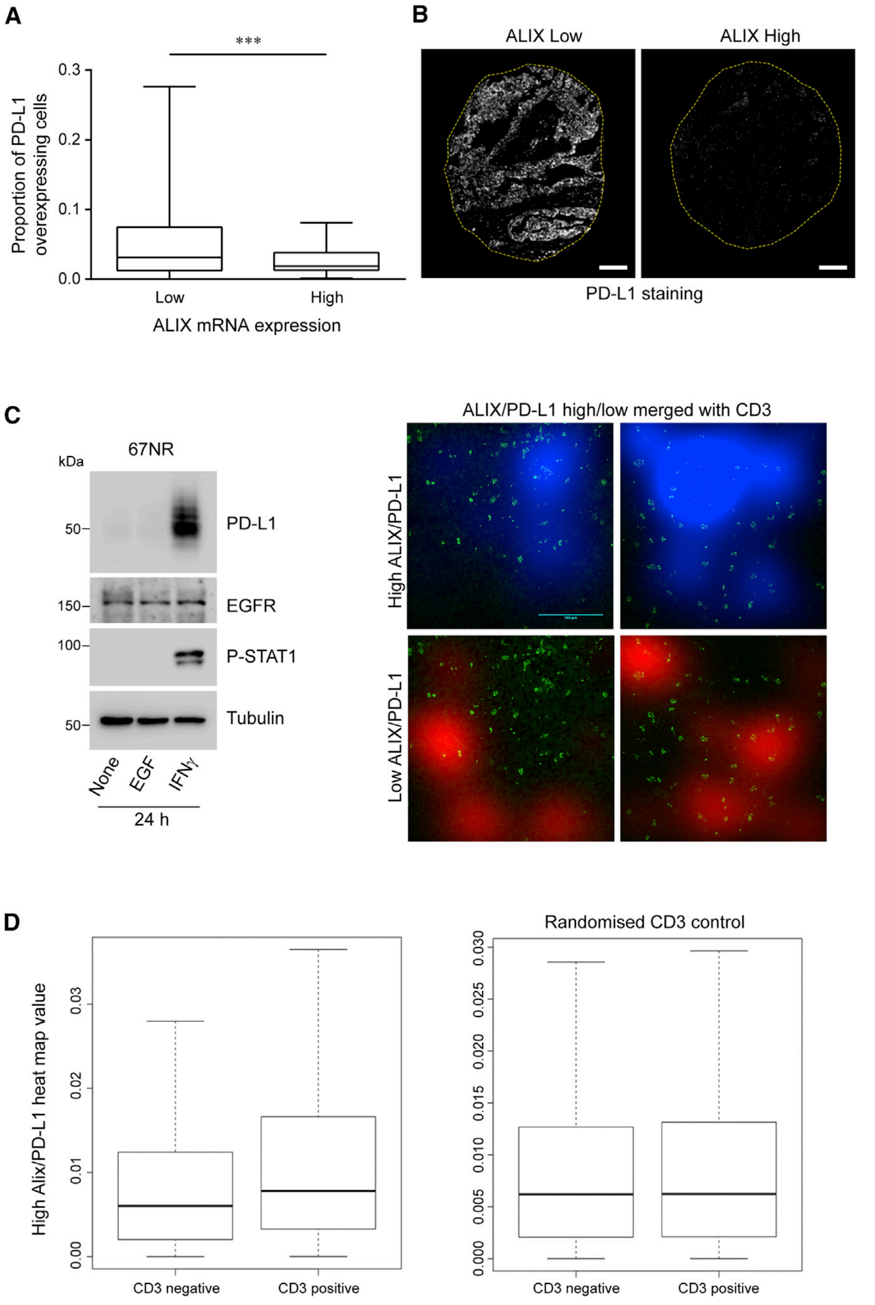

Figure 6. *In Vivo* Relationship between ALIX and PD-L1 Expression and Its Association with Immune Cell Infiltrate

(A) Boxplot of PD-L1 protein expression according to "low" (n = 95) or "high" (n = 94) ALIX mRNA expression, where "low" and "high" indicate less than and greater than the median value, respectively (***$p < 0.001$, Mann-Whitney U, two-tailed test).

(B) Fluorescence images of PD-L1 immunostaining of representative breast cancer tissue specimens associated with low (left) and high (right) ALIX mRNA expression (dotted line indicates the perimeter of tissue specimen). Scale bars, 100 μm.

(C) Western blot showing IFNγ-dependent induction of PD-L1 in 67NR cells. Spatial analysis of ALIX, PD-L1, and CD3 expression in tissue sections processed for analysis by immunofluorescence. Comparison of CD3+ cell position with heatmaps of high and low ALIX:PD-L1 expression ratios in 67NR tumors. Representative composite images of CD3 channel (green) with ALIX:PD-L1 ratio heatmaps (red and blue). Heatmaps were generated by applying a 2D Gaussian image blur to a thresholded ALIX:PD-L1 ratio image. Ratio images were thresholded for pixel regions (>1 pixel) where the ratio > 2.0 (high ratio) or < 0.5 (low ratio) after ALIX intensity values were normalized to achieve a baseline ratio of 1.0. Pixels where the ALIX:PD-L1 ratio was <0.5 (low ratio) are more excluded for CD3+ T cell infiltration. Scale bar, 100 μm.

(D) Quantification of data from (C). Left panel: in 67NR tumors, the presence of CD3 correlates with a higher heatmap value (CD3+ mean = 0.0115, SEM = 0.0002; CD3− mean = 0.00884, SEM = 0.00005, Kolmogorov-Smirnov test, $p < 0.001$). Right panel: representative baseline correlation by randomizing the position of CD3 value (CD3+ mean = 0.00914, SEM = 0.0002; CD3− mean = 0.00903, SEM = 0.00006, Kolmogorov-Smirnov test, $p = 0.6$). A total of 12 images were analyzed. Boxplots represent 30,000 20 × 20 pixel regions.

limiting membrane. We suggest that defective ILV incorporation of PD-L1 results in impaired exosomal packaging, and upon MVB-PM (plasma membrane) fusion, this results in an elevation of cell-surface PD-L1 that confers an enhanced immunosuppressive phenotype. It should be noted that, given its role in viral budding, ALIX may also contribute to the formation of plasma membrane microvesicles, which could contribute to the PD-L1 cell-surface phenotype. However, our immunocytochemistry data indicate that defective trafficking (limiting membrane to ILV) of PD-L1 within MVBs is likely to be a major contributor to this phenotype.

Analysis of tissue microarrays from diverse human breast cancer subtypes revealed PD-L1 upregulation in tissues with low ALIX mRNA levels. Previous studies observed that PD-L1 expression is heterogeneous among different breast cancers and has a prognostic and predictive value in breast cancers (Sabatier et al., 2015). Among breast cancer subtypes, BLBC cells constitutively express the highest levels of PD-L1 (Soliman et al., 2014). Furthermore, PD-L1 overexpression is more prevalent in inflammatory breast cancers (a rare and particularly aggressive form of disease) that are ER (estrogen receptor) negative, basal, and ERBB2 enriched (Bertucci et al., 2015). Our data confirm that PD-L1 expression is higher in basal-like cancers and show that PD-L1 is upregulated in tumor tissues with low ALIX mRNA levels, independently of cancer subtype.

OPEN ACCESS
CellPress

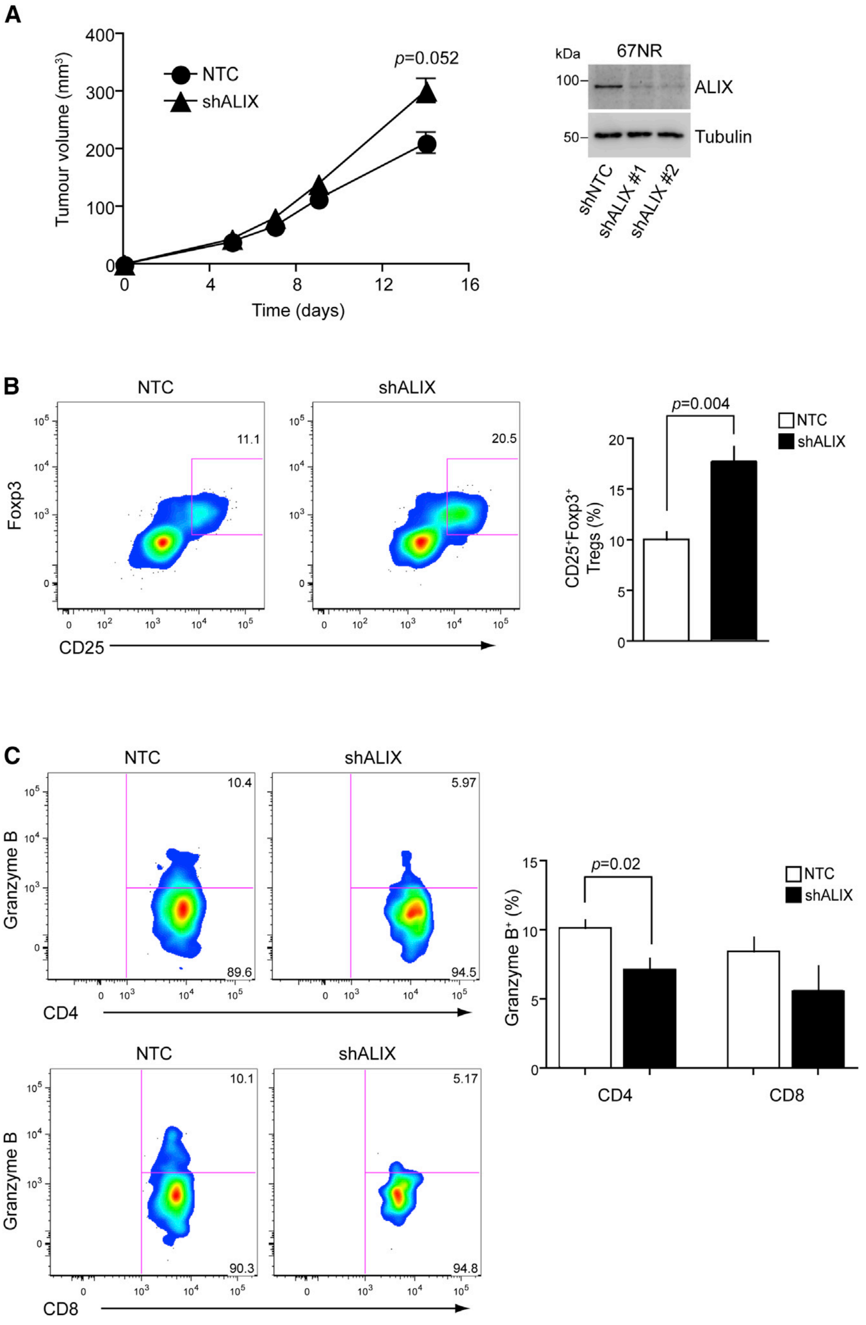

Figure 7. ALIX Deficiency in an *In Vivo* Model of Breast Cancer Induces an Immunosuppressive Tumor Microenvironment

(A) Breast tumors were induced by bilateral subcutaneous injection with control (black circle) or ALIX-KD (black triangle) 67NR cells and were monitored for up to 14 days. Western blot demonstrates stable KD of ALIX in 67NR cell lines.

(B and C) Tumor immune cell infiltrate was analyzed for the presence of regulatory T cells (B; Treg, CD4+CD25+Foxp3+) and cytotoxic T cells (C; Granzyme B expression). Plots shown are derived from gates for live CD45+CD3+ cells. The results in the three panels are representative of 8 NTC and 16 short hairpin (sh)ALIX tumors growing in 4 and 9 mice, respectively. Data for the two stable ALIX shRNA 67NR cell lines were pooled for the analysis.

Ex vivo quantitative immunofluorescence analysis of 67NR mouse tumor tissue sections revealed an intratumoral association between areas of low ALIX:PD-L1 expression ratio and a reduced CD3+ lymphocytic infiltration. While this association was significant, it should be noted that it was not observed uniformly, and it is likely that analysis was confounded by the presence of non-tumor cell types expressing ALIX and PD-L1. Spectral deconvolution of more cell-type-specific fluorescent markers will be required to delineate further this phenomenon of relative T cell exclusion from low-ALIX, high-PD-L1 tumor cells.

The generation of stable ALIX-suppressed 67NR cells enabled us to address directly the pathophysiological consequences of ALIX depletion on tumor growth and immunosuppression. 67NR cells can be used with immunocompetent BALB/c mice as a syngeneic model of breast cancer and provide an ideal tool to study tumor–immune interplay in this disease. Our *in vivo* studies revealed that ALIX-KD tumors were both larger than control tumors and associated with an enhanced immunosuppressive phenotype that included elevated numbers of infiltrating T-regulatory cells and decreased numbers of granzyme-B-expressing, tumor-infiltrating CD4+ and CD8+ T cells.

Taken together, our data strengthen the link between the tumor microenvironment and suppression of the immune system in human breast cancers by shedding light on the signaling interplay between EGFR, ALIX, and PD-L1. These data will open additional avenues for therapeutic strategies and accelerate biomarker discovery programs to optimize future combinations between EGFR and immune checkpoint targeting.

EXPERIMENTAL PROCEDURES

Antibodies

Details of antibodies used in this study are provided in Table S4.

Cell Culture

Human HCC1954 and SKBR3 breast cancer cells were cultured in RPMI 1640 and DMEM, respectively. Mouse 67NR breast and KPB6 lung cancer cells were cultured in DMEM and IMEM, respectively. Culture media was supplemented

with 10% fetal bovine serum (FBS), 2 mM L-glutamine, 100 U/mL penicillin, and 100 μg/mL streptomycin. Cell lines stably expressing NTC and ALIX shRNAs were generated by lentiviral transduction using the pGIPZ system (GE Healthcare UK, Buckinghamshire, UK). Stable cultures of virally traduced cells were established by puromycin selection.

High-Content siRNA Screen

The custom-designed Silencer Select Human siRNA Library, comprising siRNAs for 533 human gene targets (three siRNAs per target), was from Applied Biosystems. siRNA dilutions, transfection complex preparation, and cell seeding were performed using a Janus automated workstation (PerkinElmer). Each siRNA was evaluated in triplicate, and each experiment was performed three times. Cells were seeded in the presence of transfection complexes (0.5 pmol siRNA; RNAiMAX reagent, Invitrogen, Carlsbad, CA, USA) and, after 24 hr, were transfected with the Picchu-X biosensor using FuGENE HD reagent (Promega, Fitchburg, WI, USA). After an additional 24 hr, cells were treated with or without 100 ng/mL EGF for 30 min before fixation and FLIM analysis. Control siRNA cells were included alongside target siRNA cells to enable pairwise comparisons. siRNAs against human *PDCD6IP* were purchased from Horizon Discovery (Cambridge, UK). *PDCD6IP* siRNAs #1 and #2 refer to siGENOME IDs D-004233-01-0002 and D-004233-06-0002, respectively. siGENOME Non-Targeting siRNA ID D-001210-02-05 was used as the control. Lentiviral shRNA vectors for the stable KD of human and mouse *PDCD6IP* were purchased from GE Healthcare UK. For stable KD of human *PDCD6IP*, Clone ID V2LHS_357889 was used. For stable KD of murine *Pdcd6ip*, Clone IDs V2LMM_177842 and V3LMM_450035 were used.

FLIM Analysis

FLIM was used to measure FRET between the donor and acceptor fluorophores of the biosensor in cells treated with or without EGF. Because high biosensor expression increases the probability of *inter*molecular FRET between the donor and acceptor fluorophores of neighboring proteins through molecular crowding, such effects were considered in the analysis of *intra*molecular FRET (desired component). Intermolecular FRET was determined by analyzing the lifetime of the donor fluorophore at increasing concentrations of biosensor expression (determined by fluorescence intensity). Pearson correlation was used to examine the correlation between lifetime and intensity. Regression analyses were further used to quantify associations between EGF treatment, intensity, and lifetime. Tests for an interaction incorporating the product term of EGF treatment and intensity were performed to assess whether effects of EGF on lifetime differed by intensity. A lack of significant interaction indicated that the association between lifetime and EGF treatment was not affected by intensity (Figure 1E). Analyses of covariance (ANCOVA) were performed to further discern the effect of EGF treatment, with intensity and EGF treatment as predictor variables and biosensor lifetime as the dependent variable. Lifetime and intensity data were normally distributed. All statistical analyses were performed with R v3.1.2 (R Project for Statistical Computing, Vienna, Austria). Two-tailed p values < 0.05 were considered statistically significant. In all cases, data are presented as mean ± SEM.

Construction of the Picchu-X Biosensor

The Picchu-X sensor for reporting EGFR kinase activity was a kind gift from M. Matsuda, Osaka University, Osaka, Japan (Kurokawa et al., 2001). The CrkII-based sensing region was excised and inserted into the equivalent sensing portion of the previously described Raichu-Rac 1011-X sensor (Vega et al., 2011), resulting in the generation of the EGFP-CrkII-mRFP1 biosensor with a C-terminal membrane-targeting CAAX motif.

Exosome Isolation

HCC1954 cells at approximately 80% confluency were washed with PBS and then cultured for 24 hr in FBS-free media. Culture supernatants were then collected, and exosomes were enriched by sequential centrifugation. Briefly, supernatants were centrifuged at 300 × *g* for 10 min to remove cell debris, at 5,000 × *g* for 20 min to remove large vesicles and membrane fragments, at 12,200 × *g* for 60 min to deplete MVs, and then at 100,000 × *g* for 120 min to pellet exosomes. The pellet was washed in PBS and then centrifuged at 100,000 × *g* for an additional 60 min before resuspension in PBS. All steps were performed at 4°C. NTA was performed using a Nanosight LM10-HS (Nanosight) as described previously (Dragovic et al., 2011), using constant flow injection. The NTA analysis software was used to obtain information regarding the particle population size distribution including the derivation of the population's modal particle diameter. Samples for mass spectrometry were prepared and analyzed as described previously (Turriziani et al., 2014). Further details of the procedure are provided in the Supplemental Experimental Procedures.

Immunoblotting Assays

Western blotting was performed according to standard protocols. Dot-blotting was used to examine protein content of EVs due to its improved sensitivity compared with western blotting and involved the immunodetection of proteins spotted onto nitrocellulose membranes (1 μg total protein in 5 μL). All primary antibodies used are listed in Table S4. Densitometry analysis of protein band (western blots) and spot (dot-blotting) intensities was performed using ImageJ software. Data reported in bar graphs were obtained from ≥3 independent experiments.

TEM

TEM was performed on a Joel 1010 electron microscope (Joel, Warwickshire, UK). Exosomes were layered onto Formvar carbon-coated copper grids. Grids were washed with H_2O, and then incubated with 2% uranyl acetate (negative stain) for 30 s. For immunogold labeling, exosomes layered onto Formvar carbon-coated nickel grids were fixed with 0.5% paraformaldehyde (PFA) for 4 min and then blocked with serum at room temperature for 10 min. Exosomes were stained overnight at 4°C with primary antibody against human CD63, washed three times in PBS for 10 min, and subsequently labeled with secondary antibody (10 nm colloidal gold, BioCell) at room temperature for 90 min. The immunolabeled exosomes were washed with PBS for 5 min, fixed in 1% glutaraldehyde, and then washed twice with PBS and once with H_2O. Exosomes were negatively stained as described earlier before imaging.

Confocal Imaging

HCC1954 cells were transfected with ALIX siRNA (40 nM) using RNAiMAX. After 48 hr, cells were treated with IFNγ (20 ng/mL) for 24 hr. Cells were fixed with PFA and stained for CD63 and PD-L1. Nuclei were counterstained with DAPI. Cells were imaged on a spinning disk confocal microscope (Nikon Eclipse, teamed with the CSU-X1 Andor Spinning Disk with Neo sCMOS camera), acquiring sub-saturated images as z stacks with 0.3-μm Z-spacing. At least 10 fields of view per treatment were captured, and luminal versus limiting membrane staining of endosomal PD-L1 was scored by manually scrolling through Z.

Tumor Cell-T Cell Co-culture Assays

Peripheral blood mononuclear cells (PBMCs) from healthy donor blood were collected by sucrose gradient centrifugation using Ficoll (GE Healthcare). $CD3^+/CD25^-$ cells were subsequently isolated using magnetic beads (Miltenyi Biotec), labeled with eFluor 450 cell-tracker dye, and then activated with CD3, CD2, and CD28 beads at the time of co-culture with tumor cells. At 96 hr post-co-culture, cells were recovered and stained with CD3, CD4, CD8, and PD-1 antibodies and then subjected to flow cytometry. Data were analyzed using FlowJo software (FlowJo, Ashland, OR, USA). The significance of statistical differences between treatment groups was determined using ANOVA ($p < 0.05$ was reported as significant).

In Vivo Breast Cancer Model

Experiments were performed in accordance with the UK Home Office Animals Scientific Procedures Act, 1986, and the United Kingdom Co-ordinating Committee on Cancer Research (UKCCCR) guidelines. Tumors were established by subcutaneous injection of 1×10^6 cells of the syngeneic mouse breast cancer cell line 67NR (Aslakson and Miller, 1992) into the mammary fat pad of 6- to 8-week-old BALB/c female mice (Charles River UK). Tumor growth was monitored with calipers and recorded every other day for 2 weeks; then, mice were culled and primary tumors were dissected. Tumors were minced and incubated for 1 hr at 37°C in digestion media (RPMI containing 1 mg/mL type 2 collagenase and 0.1 mg/mL bovine pancreas DNase I) and passed through a 40-μm cell strainer to form single-cell suspensions for flow cytometry.

Flow Cytometry

Cell suspensions were stained with Zombie Aqua dye, followed by membrane staining with anti-CD45-AF700, CD3-PECy7, CD4-BV785, CD8-PerCpCy5.5, and CD25-PE; fixation and permeabilization; and intracellular staining with anti-Foxp3-eFluor 450 or Granzyme B-APC. Stained cell suspensions were analyzed by flow cytometry. Data were acquired in a Fortessa II flow cytometer (BD Biosciences) and analyzed with FlowJo software (FlowJo).

Patient Samples and Immunohistochemistry

Tissues and data were collected by the KHP Cancer Biobank with approval from the East of England – Cambridge East Research Ethics Committee, reference number 12/EE/0493. Tissue microarrays (TMAs) of 218 primary breast cancers from the METABRIC study (Curtis et al., 2012) were used in this analysis. After antigen retrieval using the Ventana BenchMark system (Ventana Medical Systems), tissues were stained with anti-PD-L1 antibody. A total of 189 TMA cores were imaged using an "open" high-content microscope (Barber et al., 2013), and image data were subsequently processed to determine protein expression. To find the percentage of PD-L1-positive cancer cells, we adopted a previously reported algorithm (Camp et al., 2002; Moeder et al., 2009; Wimberly et al., 2015).

For the evaluation of ALIX mRNA expression, Illumina HT-12 microarray expression data were obtained from all METABRIC samples and filtered to remove arrays with outlying low intensity (mean $\log_2$ expression < 5.6). Array data were then quantile-normalized, filtered for probe detection (required $p < 0.01$ for >1% of King's METABRIC samples), and COMBAT-corrected for beadchips. Normalization was performed using the "beadarray" package for R/Bioconductor (Dunning et al., 2007). ALIX (*PDCD6IP*) mRNA expression was inspected using Probe identifier ILMN_1693259. Gene expression data from the METABRIC study have been reported previously (Curtis et al., 2012). The correlation between PD-L1 positivity and ALIX mRNA tissue expression was tested using GraphPad statistical analysis software.

Tissue samples and data from patients were obtained from The King's Health Partners (KHP) Cancer Biobank at Guy's Hospital (London, UK; REC no.: 07/40874/131).

SUPPLEMENTAL INFORMATION

Supplemental Information includes Supplemental Experimental Procedures, six figures, and four tables and can be found with this article online at https://doi.org/10.1016/j.celrep.2018.06.066.

ACKNOWLEDGMENTS

The authors thank Pascale Zimmermann for discussion and advice on ALIX signaling and exosome biology. We also thank Susanne Heck, Anna Rose, and Rianne Wester in the Biomedical Research Center, Flow Cytometry Research Platform, Guy's Hospital, London, and the Nikon Imaging Centre at King's College London. This work was supported by Cancer Research UK (grants C1519/A6906 and C5255/A15935); by the King's College London-UCL Comprehensive Cancer Imaging Centre (CRUK and EPSRC, grants C1519/A10331 and C1519/A16463), in association with the MRC and DoH (grant C1519/A10331); by the EU FP7 grants IMAGINT (EC grant: 259881); and by KCL Breast Cancer Now Unit funding (for F.F.-B.; grant KCL-Q2-Y5). J.G.C. is a Wellcome Trust Senior Research fellow (206346/Z/17/Z). The purchase of the siRNA library was possible thanks to a generous gift from Ms. Marianne B. Blake, who raised funds during the 2010 London Marathon through Dimbleby Cancer Care.

AUTHOR CONTRIBUTIONS

T.N. conceived, supervised, and secured funding for the project. J.M., H.M., F.F.-B., G.W., and J.G.C. conceived and conducted experiments. A.C., R.C., T.B., and A.A. conducted experiments. K.L., P.R.B., W.W., and A.V.K. conducted data analysis. J.M.V., M.K., N.W., A.V.K., M.T., D.F., and B.V. provided technical expertise. C.G. and S.M.D. provided resources and expertise. S.A.Q., F.F., C.E.F., and W.K. provided expertise and intellectual feedback. T.N., J.G.C., and J.M. wrote the manuscript.

DECLARATION OF INTERESTS

The authors declare no competing interests.

Received: October 17, 2017
Revised: April 23, 2018
Accepted: June 15, 2018
Published: July 17, 2018

REFERENCES

Ahn, M.J., Yang, J., Yu, H., Saka, H., Ramalingam, S., Goto, K., Kim, S.W., Yang, L., Walding, A., and Oxnard, G.R. (2016). 136O: Osimertinib combined with durvalumab in EGFR-mutant non-small cell lung cancer: Results from the TATTON phase Ib trial. J. Thorac. Oncol. *11* (*4, Suppl*), S115.

Akbay, E.A., Koyama, S., Carretero, J., Altabef, A., Tchaicha, J.H., Christensen, C.L., Mikse, O.R., Cherniack, A.D., Beauchamp, E.M., Pugh, T.J., et al. (2013). Activation of the PD-1 pathway contributes to immune escape in EGFR-driven lung tumors. Cancer Discov. *3*, 1355–1363.

Aslakson, C.J., and Miller, F.R. (1992). Selective events in the metastatic process defined by analysis of the sequential dissemination of subpopulations of a mouse mammary tumor. Cancer Res. *52*, 1399–1405.

Azuma, K., Ota, K., Kawahara, A., Hattori, S., Iwama, E., Harada, T., Matsumoto, K., Takayama, K., Takamori, S., Kage, M., et al. (2014). Association of PD-L1 overexpression with activating EGFR mutations in surgically resected nonsmall-cell lung cancer. Ann. Oncol. *25*, 1935–1940.

Baietti, M.F., Zhang, Z., Mortier, E., Melchior, A., Degeest, G., Geeraerts, A., Ivarsson, Y., Depoortere, F., Coomans, C., Vermeiren, E., et al. (2012). Syndecan-syntenin-ALIX regulates the biogenesis of exosomes. Nat. Cell Biol. *14*, 677–685.

Barber, P.R., Tullis, I.D., Pierce, G.P., Newman, R.G., Prentice, J., Rowley, M.I., Matthews, D.R., Ameer-Beg, S.M., and Vojnovic, B. (2013). The Gray Institute 'open' high-content, fluorescence lifetime microscopes. J. Microsc. *251*, 154–167.

Bertucci, F., Finetti, P., Colpaert, C., Mamessier, E., Parizel, M., Dirix, L., Viens, P., Birnbaum, D., and van Laere, S. (2015). PDL1 expression in inflammatory breast cancer is frequent and predicts for the pathological response to chemotherapy. Oncotarget *6*, 13506–13519.

Bissig, C., and Gruenberg, J. (2014). ALIX and the multivesicular endosome: ALIX in wonderland. Trends Cell Biol. *24*, 19–25.

Burness, M.L., Grushko, T.A., and Olopade, O.I. (2010). Epidermal growth factor receptor in triple-negative and basal-like breast cancer: promising clinical target or only a marker? Cancer J. *16*, 23–32.

Camp, R.L., Chung, G.G., and Rimm, D.L. (2002). Automated subcellular localization and quantification of protein expression in tissue microarrays. Nat. Med. *8*, 1323–1327.

Carey, L.A., Rugo, H.S., Marcom, P.K., Mayer, E.L., Esteva, F.J., Ma, C.X., Liu, M.C., Storniolo, A.M., Rimawi, M.F., Forero-Torres, A., et al. (2012). TBCRC 001: randomized phase II study of cetuximab in combination with carboplatin in stage IV triple-negative breast cancer. J. Clin. Oncol. *30*, 2615–2623.

Carlton, J. (2010). The ESCRT machinery: a cellular apparatus for sorting and scission. Biochem. Soc. Trans. *38*, 1397–1412.

Chen, N., Fang, W., Zhan, J., Hong, S., Tang, Y., Kang, S., Zhang, Y., He, X., Zhou, T., Qin, T., et al. (2015). Upregulation of PD-L1 by EGFR activation mediates the immune escape in EGFR-driven NSCLC: implication for optional immune targeted therapy for NSCLC patients with EGFR mutation. J. Thorac. Oncol. *10*, 910–923.

Chong, C.R., and Jänne, P.A. (2013). The quest to overcome resistance to EGFR-targeted therapies in cancer. Nat. Med. *19*, 1389–1400.

Ciardiello, F., and Tortora, G. (2008). EGFR antagonists in cancer treatment. N. Engl. J. Med. *358*, 1160–1174.

Curtis, C., Shah, S.P., Chin, S.F., Turashvili, G., Rueda, O.M., Dunning, M.J., Speed, D., Lynch, A.G., Samarajiwa, S., Yuan, Y., et al.; METABRIC Group (2012). The genomic and transcriptomic architecture of 2,000 breast tumours reveals novel subgroups. Nature *486*, 346–352.

Dragovic, R.A., Gardiner, C., Brooks, A.S., Tannetta, D.S., Ferguson, D.J., Hole, P., Carr, B., Redman, C.W., Harris, A.L., Dobson, P.J., et al. (2011). Sizing and phenotyping of cellular vesicles using nanoparticle tracking analysis. Nanomedicine (Lond.) *7*, 780–788.

Dunning, M.J., Smith, M.L., Ritchie, M.E., and Tavaré, S. (2007). beadarray: R classes and methods for Illumina bead-based data. Bioinformatics *23*, 2183–2184.

Ghossoub, R., Lembo, F., Rubio, A., Gaillard, C.B., Bouchet, J., Vitale, N., Slavík, J., Machala, M., and Zimmermann, P. (2014). Syntenin-ALIX exosome biogenesis and budding into multivesicular bodies are controlled by ARF6 and PLD2. Nat. Commun. *5*, 3477.

Hashimoto, Y., Katayama, H., Kiyokawa, E., Ota, S., Kurata, T., Gotoh, N., Otsuka, N., Shibata, M., and Matsuda, M. (1998). Phosphorylation of CrkII adaptor protein at tyrosine 221 by epidermal growth factor receptor. J. Biol. Chem. *273*, 17186–17191.

Hudis, C.A., and Gianni, L. (2011). Triple-negative breast cancer: an unmet medical need. Oncologist *16* (*Suppl 1*), 1–11.

Irshad, S., Ellis, P., and Tutt, A. (2011). Molecular heterogeneity of triple-negative breast cancer and its clinical implications. Curr. Opin. Oncol. *23*, 566–577.

Komatsu, N., Aoki, K., Yamada, M., Yukinaga, H., Fujita, Y., Kamioka, Y., and Matsuda, M. (2011). Development of an optimized backbone of FRET biosensors for kinases and GTPases. Mol. Biol. Cell *22*, 4647–4656.

Kurokawa, K., Mochizuki, N., Ohba, Y., Mizuno, H., Miyawaki, A., and Matsuda, M. (2001). A pair of fluorescent resonance energy transfer-based probes for tyrosine phosphorylation of the CrkII adaptor protein in vivo. J. Biol. Chem. *276*, 31305–31310.

Masuda, H., Zhang, D., Bartholomeusz, C., Doihara, H., Hortobagyi, G.N., and Ueno, N.T. (2012). Role of epidermal growth factor receptor in breast cancer. Breast Cancer Res. Treat. *136*, 331–345.

Moeder, C.B., Giltnane, J.M., Moulis, S.P., and Rimm, D.L. (2009). Quantitative, fluorescence-based in-situ assessment of protein expression. Methods Mol. Biol. *520*, 163–175.

Moon, P.G., Lee, J.E., You, S., Kim, T.K., Cho, J.H., Kim, I.S., Kwon, T.H., Kim, C.D., Park, S.H., Hwang, D., et al. (2011). Proteomic analysis of urinary exosomes from patients of early IgA nephropathy and thin basement membrane nephropathy. Proteomics *11*, 2459–2475.

Murillo, M.M., Zelenay, S., Nye, E., Castellano, E., Lassailly, F., Stamp, G., and Downward, J. (2014). RAS interaction with PI3K p110α is required for tumor-induced angiogenesis. J. Clin. Invest. *124*, 3601–3611.

Nielsen, T.O., Hsu, F.D., Jensen, K., Cheang, M., Karaca, G., Hu, Z., Hernandez-Boussard, T., Livasy, C., Cowan, D., Dressler, L., et al. (2004). Immunohistochemical and clinical characterization of the basal-like subtype of invasive breast carcinoma. Clin. Cancer Res. *10*, 5367–5374.

Nuciforo, P., Radosevic-Robin, N., Ng, T., and Scaltriti, M. (2015). Quantification of HER family receptors in breast cancer. Breast Cancer Res. *17*, 53.

Pardoll, D.M. (2012). The blockade of immune checkpoints in cancer immunotherapy. Nat. Rev. Cancer *12*, 252–264.

Sabapatha, A., Gercel-Taylor, C., and Taylor, D.D. (2006). Specific isolation of placenta-derived exosomes from the circulation of pregnant women and their immunoregulatory consequences. Am. J. Reprod. Immunol. *56*, 345–355.

Sabatier, R., Finetti, P., Mamessier, E., Adelaide, J., Chaffanet, M., Ali, H.R., Viens, P., Caldas, C., Birnbaum, D., and Bertucci, F. (2015). Prognostic and predictive value of PDL1 expression in breast cancer. Oncotarget *6*, 5449–5464.

Soliman, H., Khalil, F., and Antonia, S. (2014). PD-L1 expression is increased in a subset of basal type breast cancer cells. PLoS ONE *9*, e88557.

Turriziani, B., Garcia-Munoz, A., Pilkington, R., Raso, C., Kolch, W., and von Kriegsheim, A. (2014). On-beads digestion in conjunction with data-dependent mass spectrometry: a shortcut to quantitative and dynamic interaction proteomics. Biology (Basel) *3*, 320–332.

Vega, F.M., Fruhwirth, G., Ng, T., and Ridley, A.J. (2011). RhoA and RhoC have distinct roles in migration and invasion by acting through different targets. J. Cell Biol. *193*, 655–665.

Wimberly, H., Brown, J.R., Schalper, K., Haack, H., Silver, M.R., Nixon, C., Bossuyt, V., Pusztai, L., Lannin, D.R., and Rimm, D.L. (2015). PD-L1 expression correlates with tumor-infiltrating lymphocytes and response to neoadjuvant chemotherapy in breast cancer. Cancer Immunol. Res. *3*, 326–332.

Cell Reports, Volume *24*

Supplemental Information

ALIX Regulates Tumor-Mediated Immunosuppression by Controlling EGFR Activity and PD-L1 Presentation

James Monypenny, Hanna Milewicz, Fabian Flores-Borja, Gregory Weitsman, Anthony Cheung, Ruhe Chowdhury, Thomas Burgoyne, Appitha Arulappu, Katherine Lawler, Paul R. Barber, Jose M. Vicencio, Melanie Keppler, Wahyu Wulaningsih, Sean M. Davidson, Franca Fraternali, Natalie Woodman, Mark Turmaine, Cheryl Gillett, Dafne Franz, Sergio A. Quezada, Clare E. Futter, Alex Von Kriegsheim, Walter Kolch, Borivoj Vojnovic, Jeremy G. Carlton, and Tony Ng

Supplemental figures

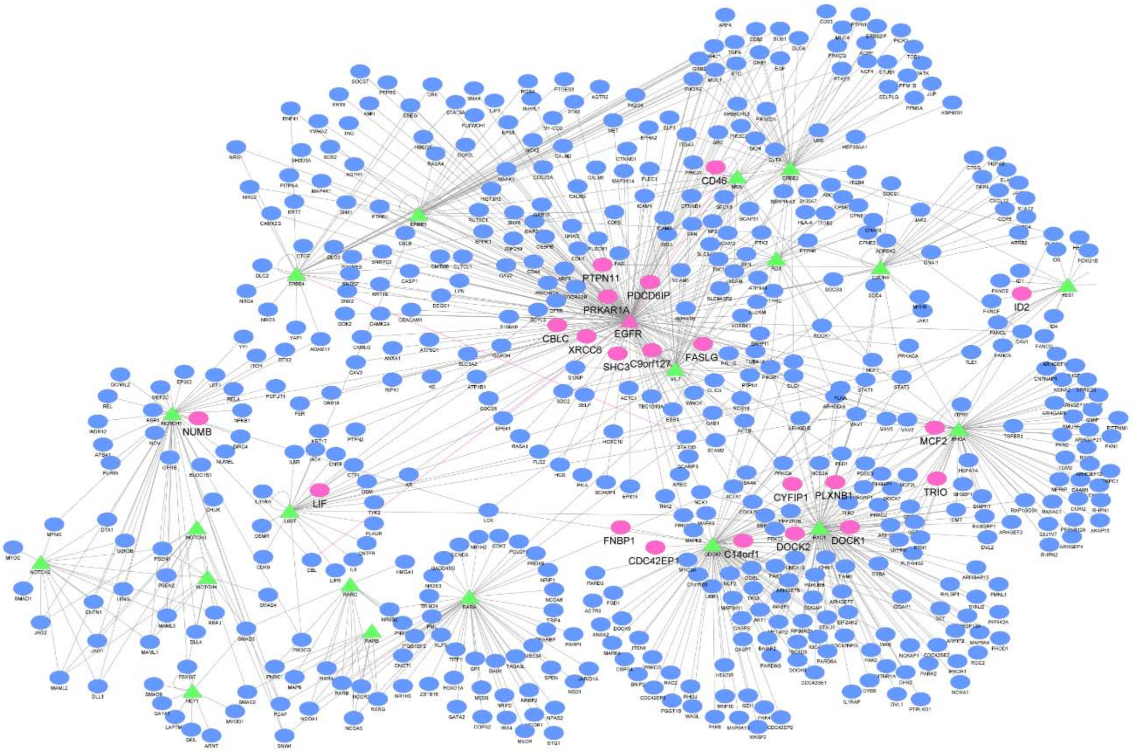

Figure S1. The EGFR protein interaction network, Related to Figure 2.

Visual representation of the EGFR protein interaction network generated using Cytoscape software (The Cytoscape Consortium: http://www.cytoscape.org/). The network was designed using the Human Protein Reference Database (HPRD, http://www.hprd.org/) to predict potential targets that contribute to a malignant phenotype in breast cancer. The HPRD was mined for proteins within an EGFR-containing protein subnetwork which included the EGFR family members (EGFR, HER2, HER3 and HER4), as well 16 other seed proteins from our previously published sub-network constituting membrane receptors, protein kinases, and cytoskeletal proteins associated with a cancer 'metastatic' phenotype (Fruhwirth et al., 2011; Weitsman et al., 2014). These proteins were used as seed-set proteins (green triangles), which together with their interacting partners, may contain novel regulators of the EGFR signaling network. Proteins were extracted from the HPRD that interact directly with at least one of the seed set-proteins as well as a second-degree interactor (i.e. partner of the partners). A total of 533 proteins that satisfied these criteria were extracted from the database (blue and pink nodes), and a library of siRNAs targeting each candidate was constructed and subsequently used for the high-content Picchu-FLIM screen. Pink nodes represent proteins that were identified as hits from the siRNA screen, and therefore represent cases where protein knockdown resulted in a change in EGFR activity as measured by FRET-FLIM.

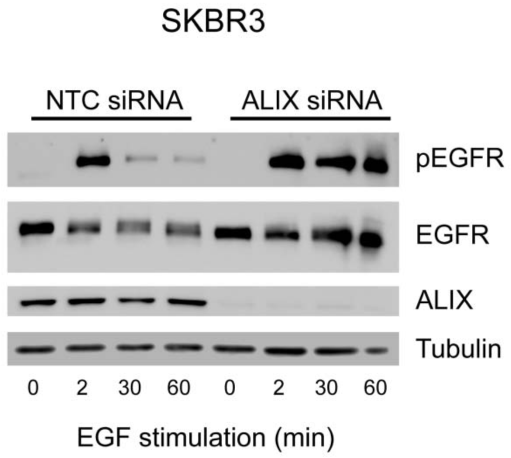

Figure S2. ALIX depletion results in sustained ligand-dependent EGFR phosphorylation in SKBR3 cells, Related to Figure 2.

Western blot of EGFR phosphorylation in NTC control and ALIX siRNA transfected SKBR3 human breast cancer cells stimulated with EGF for the indicated time points. Elevated and sustained EGFR phosphorylation is observed in ALIX knockdown cells, when compared with the NTC controls (Blot is one of two independent but similar results).

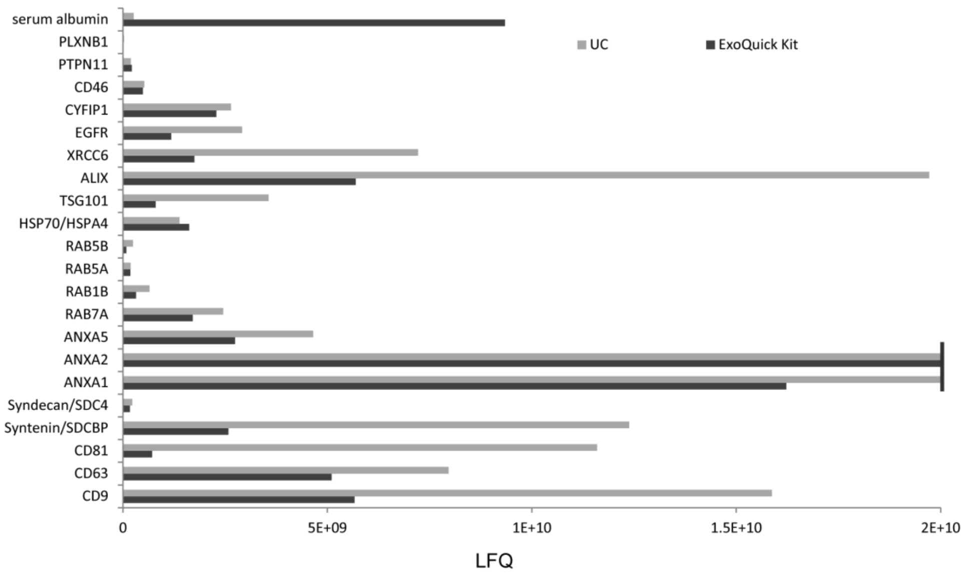

Figure S3. Comparison of proteomics data for exosome preparations obtained by ultracentrifugation and ExoQuick enrichment methods, Related to Figure 3.

Comparative proteomics analysis of HCC1954-derived exosomes isolated by ultracentrifugation (UC) or using the ExoQuick kit. Proteomics analysis reveals protein markers commonly associated with exosomes for both techniques used, although the ExioQuick method is associated with higher levels of the extracellular protein contaminant serum albumin. Exosomes were analyzed by LC-MS/MS. Relative label-free quantification (LFQ) intensities for indicated proteins are shown.

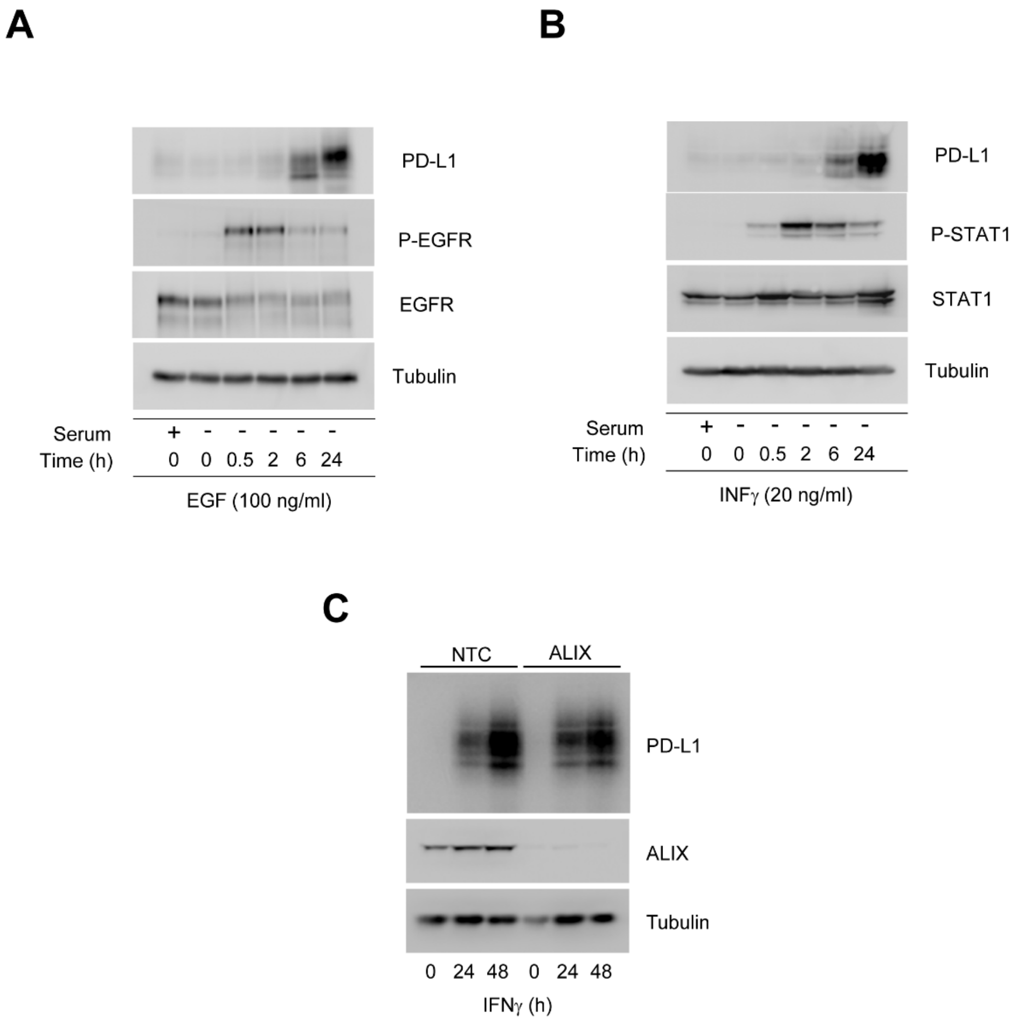

Figure S4. Ligand-dependent PD-L1 induction in HCC1954 cells, Related to Figure 4.

Representative western blots demonstrating the induction of PD-L1 protein expression in HCC1954 cells following their treatment with either EGF (A, 100 ng/ml) or IFNγ (B, 20 ng/ml). (C) Western blot showing the robust and prolonged induction of PD-L1 expression in both control and ALIX knockdown cells.

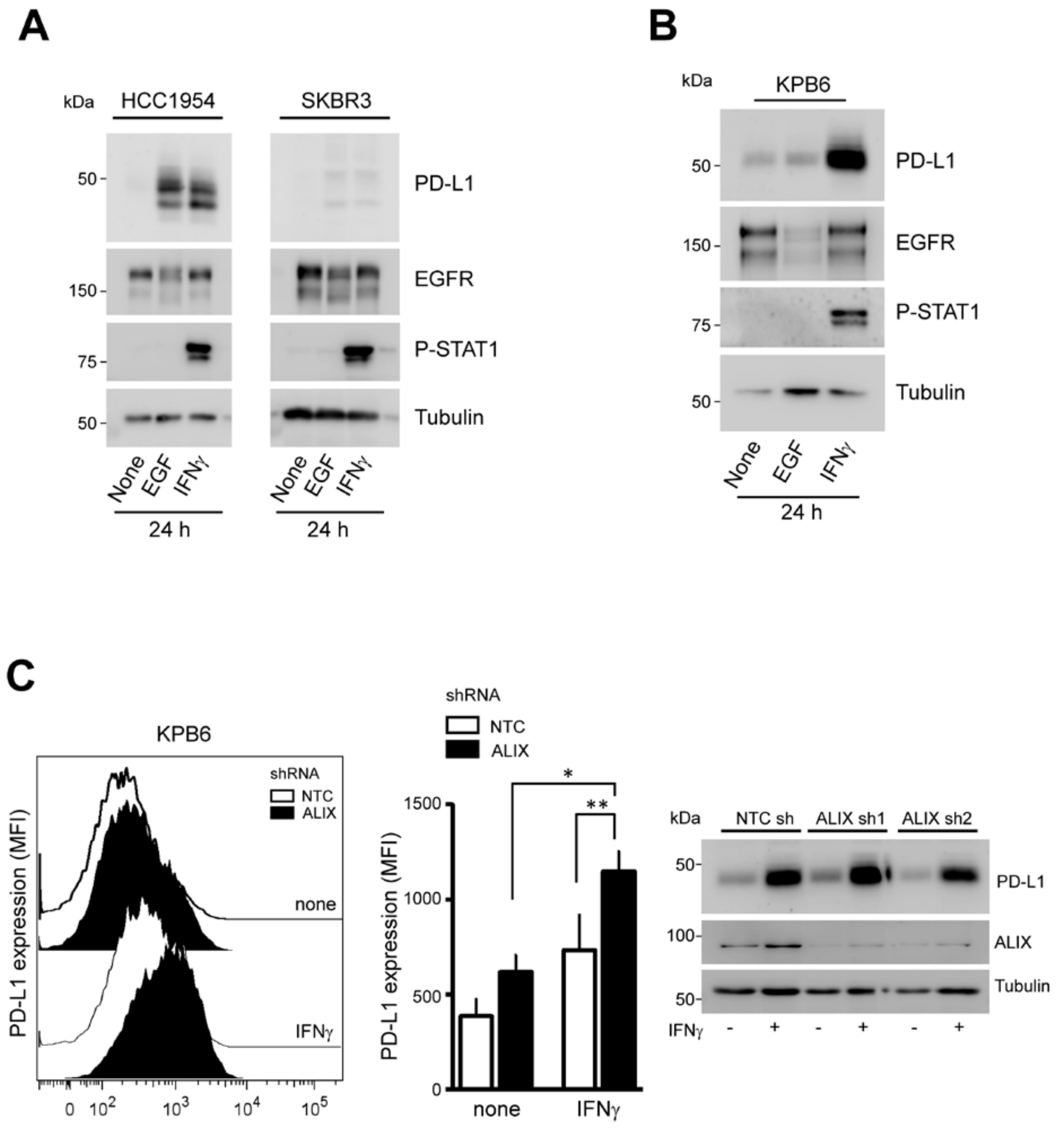

Figure S5. Ligand-dependent PD-L1 induction in various tumor cell lines, Related to Figure 5.

(A) Western blots comparing the effects of EGF and IFNγ stimulation on PD-L1 expression in HCC1954 (left column) and SKBR3 (right column) human breast carcinoma cells. Membranes for the two different cell lines were imaged side-by-side using the same exposure to enable the direct comparison of protein expression levels. (B) Western blots demonstrating the effects of EGF and IFNγ stimulation on PD-L1 expression in mouse KBP6 tumor cells. (C) Flow cytometry data (left panel) and corresponding statistical analysis (middle panel) of surface PD-L1 expression in NTC and ALIX shRNA KPB6 cells following 24 h stimulation with IFNγ. Data shown represent the pooled values of PD-L1 MFI for the two stable ALIX shRNA KPB6 cell lines used in three independent experiments. Western blot (right panel) demonstrating total PD-L1 and ALIX expression in NTC and ALIX shRNA KPB6 cells used in flow cytometry assays.

A

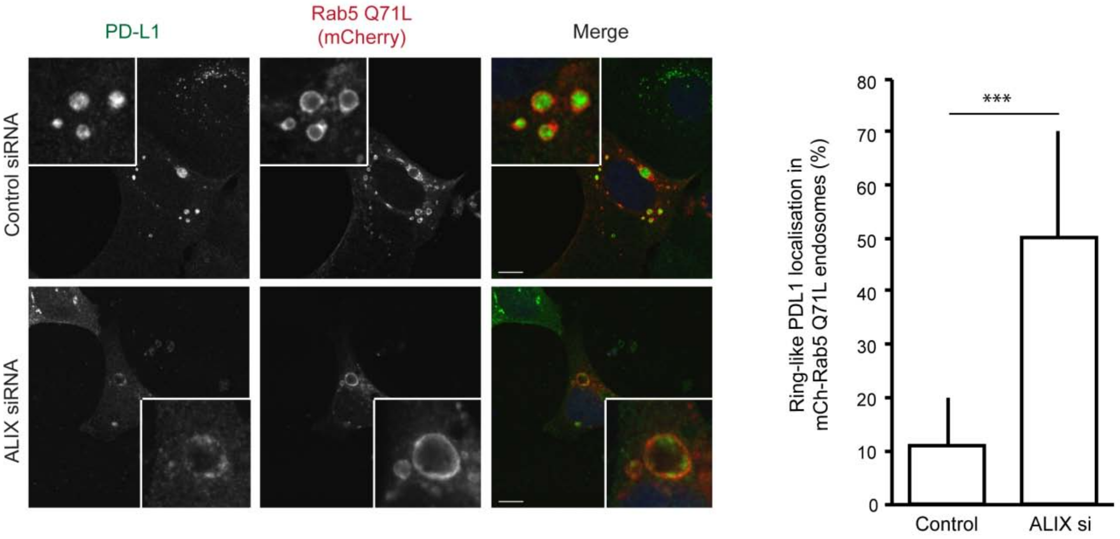

B

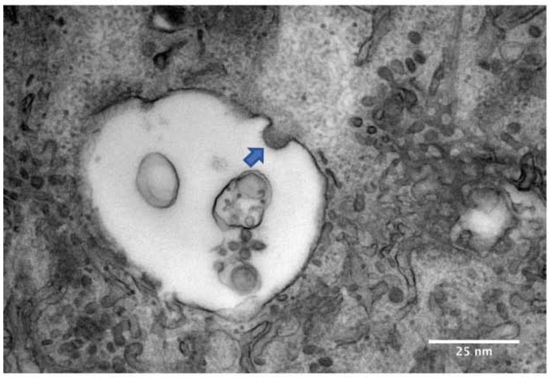

Budding profile	NTC sh	ALIX sh	
Yes	4	24	p<0.001
No	96	76	
	100	100	

Figure S6. ILV incorporation of PD-L1 is suppressed in ALIX knockdown cells, Related to Figure 5.

(A) Impaired incorporation of PD-L1 into the ILVs of ALIX knockdown cells. Confocal analysis (left-hand panel) and associated statistical analysis (right-hand bar graph) of PD-L1 localization in the MVBs of control and ALIX knockdown cells overexpressing the Rab5 Q71L mutant. IFNγ-stimulated control and ALIX siRNA cells were transfected with mCherry-tagged Rab5 Q71L plasmid and subsequently fixed and stained with anti-PD-L1 antibody. Rab5 Q71L overexpression results in the enlargement of endosomes, facilitating an analysis of PD-L1 staining within the lumen and at the limiting membrane of these structures. PD-L1 staining is largely exclude from the MVB lumen in ALIX knockdown cells and is instead confined to the limiting membrane (Control siRNA, n = 214 endosomes across 25 cells; ALIX siRNA, N = 125 endosomes across 25 cells. $P < 0.05$, two-tailed T-test). (B) Increased frequency in the observation of budding profiles in MVBs of ALIX knockdown cells. Example TEM image (left-hand panel) showing a budding profile (indicated by blue arrow) associated with an MVB of an ALIX knockdown cell. The bud, which is a precursor of an ILV, protrudes inwards towards the MVB lumen but remains attached to the limiting membrane. The presence of budding profiles was determined for 100 MVBs from cells across N = 4 independent experiments for each NTC and ALIX shRNA treatment group (right-hand table). Pooled scores were analyzed using a two-tailed Fisher's exact test.

Supplemental Tables

Category	Gene name	Gene ID	Characteristics of encoded protein
**Transmembrane proteins/receptors**	*EGFR*	1956	Transmembrane receptor tyrosine kinase
	PLXNB1	5364	Transmembrane semaphorin receptor, regulator of c-MET
	FASLG	356	Transmembrane receptor associated with apoptotic signaling
	CD46	4179	Type I transmembrane protein implicated in compliment signaling
	TMEM8B	51754	Transmembrane protein of unknown function
**Membrane receptor regulators/adaptors**	*SHC3*	53358	SH2-containing signaling adapter/docking protein
	NUMB	8650	Notch1 interacting protein/negative regulator of Notch signaling
	CBLC	23624	An E3 ubiquitin protein ligase
**Extracellular ligands**	*LIF*	3976	Pleotropic cytokine with a broad range of associated functions
**Regulators/effectors of Rho-family GTPases**	*DOCK1*	1793	Guanine nucleotide exchange factor
	MCF2	4168	Guanine nucleotide exchange factor
	TRIO	7204	Guanine nucleotide exchange factor
	CDC42EP1	11135	Effector of the Rho family GTPase Cdc42
	CYFIP1	23191	Component of the WAVE1 complex
**Non-receptor kinases**	*PRKAR1A*	5573	Regulatory subunit of type I cAMP-dependent protein kinase
**Non-receptor phosphatases**	*PTPN11*	5781	Protein tyrosine phosphatase
**Endosomal trafficking**	*PDCD6IP (ALIX)*	10015	ESCRT protein associated with EV biogenesis
**DNA binding/processing**	*XRCC6*	2547	Single-stranded DNA-dependent helicase
	ID2	3398	Inhibitor of DNA binding
**Other**	*C14orf1*	11161	Protein of unknown function

Table S1. Proteins identified as regulators of EGFR in the high-content RNAi screen, Related to Figure 2.

Table listing the characteristics and IDs of the 19 genes identified as regulators of EGFR from the high content siRNA screen. EGFR is also included for completeness because it was the positive control and identified as a hit protein in the biosensor high-content screen.

Gene name	Exosomal cargo	URL	No. of citations	ISEV score
EGFR	Yes	http://exocarta.org/gene_summary?gene_id=1956	11	32
PLXNB1	Yes	http://exocarta.org/gene_summary?gene_id=5364#211	1	4
FASLG	Yes	http://exocarta.org/gene_summary?gene_id=356	3	5
CD46	Yes	http://exocarta.org/gene_summary?gene_id=4179	7	26
TMEM8B	No			
SHC3	No			
NUMB	Yes	http://exocarta.org/gene_summary?gene_id=8650	1	4
CBLC	No			
LIF	No			
DOCK1	Yes	http://exocarta.org/gene_summary?gene_id=1793	2	8
MCF2	No			
TRIO	Yes	http://exocarta.org/gene_summary?gene_id=7204	1	3
CDC42EP1	Yes	http://exocarta.org/gene_summary?gene_id=511099	1	0
CYFIP1	Yes	http://exocarta.org/gene_summary?gene_id=23191	11	38
PRKAR1A	Yes	http://exocarta.org/gene_summary?gene_id=5573	4	13
PTPN11	Yes	http://exocarta.org/gene_summary?gene_id=5781	3	10
PDCD6IP (ALIX)	Yes	http://exocarta.org/gene_summary?gene_id=10015	36	106
XRCC6	Yes	http://exocarta.org/gene_summary?gene_id=2547	7	27
ID2	No			
C14orf1	Yes	http://exocarta.org/gene_summary?gene_id=11161	1	2

Table S2. Genes identified as regulators of EGFR are associated with the exosome cargo, Related to Figure 2.

Table listing genes identified as regulators of EGFR from the high content siRNA screen and their known association with the exosomal cargo: genes were classed as encoding exosomal proteins based on their presence in the ExoCarta database (http://exocarta.org/index.html). URLs for specific gene entries are provided in the third column. “No. of citations” refers to the number of independent studies recorded in ExoCarta for a given gene. Entries in the ExoCarta database are assessed against five criteria laid down by the ISEV (Keerthikumar et al., 2016). These criteria collectively provide an indication of how rigorous a study was in its characterization of extracellular vesicles, and therefore provides a qualitative measure of the confidence with which the vesicles studied can be considered to be *bona fide* exosomes. In the table above, the “ISEV score” is the sum of all fulfilled ISEV criteria from all annotated studies for a given gene in the ExoCarta database.

Gene name	Protein expression					
	Normal human breast tissue	Human breast cancer tissue				
		High	Medium	Low	None	Total
EGFR	None detected	0	2	1	7	10
PLXNB1	Medium	2	8	1	0	11
FASLG	None detected	0	0	0	11	11
CD46	High	0	3	7	2	12
NUMB	High	2	4	1	2	9
DOCK1	High	1	2	2	7	12
TRIO	Medium	0	4	6	1	11
CDC42EP1	Low	0	0	1	10	11
CYFIP1	Medium	0	0	9	2	11
PRKAR1A	Low	1	6	3	13	12
PTPN11	Medium	5	5	1	0	11
PDCD6IP (ALIX)	Medium	0	3	7	1	11
XRCC6	High	12	0	0	0	12
C14orf1	Low	0	1	3	7	11

Table S3. Expression profile of genes identified as regulators of EGFR in normal and cancerous breast tissues, Related to Figure 2.

Table listing genes identified as regulators of EGFR from the high content siRNA screen and their protein expression in normal human breast tissues and breast cancer tissues according to the Human Protein Atlas database (http://www.proteinatlas.org). The database contained immunohistochemical expression data for all 13 of the hit proteins associated with exosomes.

**Antibody target**	**Supplier**	**Code/clone**	**Assay**
EGFR	Cell Signaling Technology	#4267	**WB/DB**
p-EGFR	Cell Signaling Technology	#4407/53A5	**WB/DB**
CrkII	Cell Signaling Technology	#3492	**WB**
p- CrkII	Cell Signaling Technology	#3491	**WB**
STAT1	Cell Signaling Technology	#9172	**WB/DB**
p-STAT1	Cell Signaling Technology	#9167	**WB/DB**
ALIX	Cell Signaling Technology	#2171	**WB/DB**
Calnexin	Cell Signaling Technology	#2679	**DB**
PD-L1	Cell Signaling Technology	#13684	**WB/DB/IF/IHC**
CD63	GenTex	#GTX28219	**DB**
CD63	Developmental Studies Hybridoma Bank	#H5C6	**IF**
CD63	gift from Fedor Berditchevski	N/A	**IEM**
TSG101	GenTex	#GTX70255	**WB**
EpCAM	Sigma	#SAB4700423	**DB**
α-actin	Sigma	#A2668	**WB**
Tubulin	Sigma	#T6074	**WB**
CD45-AF700	BioLegend	#103127/30-F11	**FC**
CD3-PECy7	BioLegend	#100319/145-2C11	**FC**
CD4-BV785	BioLegend	#100453/GK1.5	**FC**
CD8-PerCpCy5.5	BioLegend	#100733/53-6.7	**FC**
CD25-PE	BioLegend	# 102007/PC61.5	**FC**
Granzyme B-APC	BioLegend	# 372203/QA16A02	**FC**
Foxp3-eFluor450	eBioscience	# 48-5773-82/FJK-16s	**FC**

Table S4. Table of antibodies used in this study, Related to Experimental Procedures.

The table summarizes the antibodies used in this study, including associated application(s) and product and supplier information. WB; western blotting, DB; dot-blotting, FC; flow cytometery, IF; immunofluorescence, IHC; immunohistochemistry, IEM; immuno-electron microscopy. Antibody suppliers listed include Cell Signaling Technology (Danvers, MA, USA), GenTex (Letchworth Garden City, UK), Sigma-Aldrich Company Ltd. (Dorset, England), BioLegend (London, UK), and eBioscience (San Diego, CA, USA).

Supplemental Experimental Procedures

Mass spectrometry analysis of exosomes.

Pelleted exosomes were lysed in 100 μL of 1% SDS buffer, sonicated and clarified by centrifugation. The protein concentration was determined by a BCA assay (Pierce). Approximately 40 μg of protein was processed according to the FASP protocol and reduced with 100 mM DTT for 5 min at 95 °C. SDS was removed by sequential washes using 30kDa cut-off spin columns (Sartorius). The sample was then alkylated with 55 mM iodoacetamide in 50 mM ABC for 1 h at room temperature and subsequently washed by sequential wash steps with 50 mM ABC. Then, samples were digested with trypsin (Promega) at 37°C over night. Peptides were recovered by centrifugation through the cut-off membrane. Peptide concentration was determined by 280 nM absorption. Peptides were acidified (4μg per sample) with 0.5% trifluoroacetic acid and desalted with homemade STAGE-tips (Wisniewski et al., 2009).

Tryptic peptides were separated on an Ultimate Ultra3000 chromatography system incorporating an auto sampler and analyzed using a Q-Exactive mass spectrometer (Thermo Scientific, Germany). The tryptic peptides (5 μL of each sample) were loaded on a homemade column (100 mm length, 75 μm inside diameter) packed with 1.9 μm ReprosilAQ C_{18} (Dr.Maisch, Germany) and separated by an increasing acetonitrile gradient, using a 60-min reverse-phase gradient (from 3–32 % Acetonitrile) at a flow rate of 250 nL/min, operated in positive ion mode with a capillary temperature of 320 °C, with a potential of 2300 V applied to the column. Data were acquired with the mass spectrometer operating in automatic data-dependent switching mode, selecting the 12 most intense ions prior to tandem MS (MS/MS) analysis. An ion selection limit of 8300 was applied for the counts, and selected ions were dynamically excluded for the next 40 s. Mass spectra were analyzed using the label-free quantitation MaxQuant Software package. All the samples were analyzed as two technical replicates and three biological triplicates.

Data was analyzed using the MaxQuant software package. Raw data files were searched against a human database (Uniprot HUMAN) with methionine oxidation as variable modification using a mass accuracy of 4.5 ppm and 0.01 false discovery rate (FDR) at both peptide and protein level to exclude false-positives. Each file was considered as separate in the experimental design; the replicates of each condition were grouped for the subsequent statistical analysis which was performed using the Andromeda software suite. Reverse and proteins identified solely with modified peptides were excluded. LFQ intensities were log(2) transformed. All entries were deleted if at least one sample group did not have all the values for all replicates. Results were cleaned for reverse and contaminants and a list of significant interactions was determined based on average ratio and ANOVA T-test using a protein level cut-off of 1.5- or 0.67-fold change.

Supplemental References

Fruhwirth, G.O., Fernandes, L.P., Weitsman, G., Patel, G., Kelleher, M., Lawler, K., Brock, A., Poland, S.P., Matthews, D.R., Keri, G.*, et al.* (2011). How Forster resonance energy transfer imaging improves the understanding of protein interaction networks in cancer biology. Chemphyschem : a European journal of chemical physics and physical chemistry *12*, 442-461.

Keerthikumar, S., Chisanga, D., Ariyaratne, D., Al Saffar, H., Anand, S., Zhao, K., Samuel, M., Pathan, M., Jois, M., Chilamkurti, N.*, et al.* (2016). ExoCarta: A Web-Based Compendium of Exosomal Cargo. J Mol Biol *428*, 688-692.

Weitsman, G., Lawler, K., Kelleher, M.T., Barrett, J.E., Barber, P.R., Shamil, E., Festy, F., Patel, G., Fruhwirth, G.O., Huang, L.*, et al.* (2014). Imaging tumour heterogeneity of the consequences of a

PKCalpha-substrate interaction in breast cancer patients. Biochemical Society transactions *42*, 1498-1505.
Wisniewski, J.R., Zougman, A., and Mann, M. (2009). Combination of FASP and StageTip-based fractionation allows in-depth analysis of the hippocampal membrane proteome. Journal of proteome research *8*, 5674-5678.
